# Triplanar ensemble U-Net model for white matter hyperintensities segmentation on MR images

**DOI:** 10.1016/j.media.2021.102184

**Published:** 2021-10

**Authors:** Vaanathi Sundaresan, Giovanna Zamboni, Peter M. Rothwell, Mark Jenkinson, Ludovica Griffanti

**Affiliations:** a Wellcome Centre for Integrative Neuroimaging, Oxford Centre for Functional MRI of the Brain, Nuffield Department of Clinical Neurosciences, University of Oxford, UK; b Oxford-Nottingham Centre for Doctoral Training in Biomedical Imaging, University of Oxford, UK; c Oxford India Centre for Sustainable Development, Somerville College, University of Oxford, UK; d Centre for Prevention of Stroke and Dementia, Nuffield Department of Clinical Neurosciences, University of Oxford, UK; e Dipartimento di Scienze Biomediche, Metaboliche e Neuroscienze, Universitá di Modena e Reggio Emilia, Italy; f Australian Institute for Machine Learning (AIML), School of Computer Science, The University of Adelaide, Adelaide, Australia; g Wellcome Centre for Integrative Neuroimaging, Oxford Centre for Human Brain Activity, Department of Psychiatry, University of Oxford, Oxford, UK

**Keywords:** Deep learning, White matter hyperintensities, U-Nets, Segmentation, MRI

## Abstract

White matter hyperintensities (WMHs) have been associated with various cerebrovascular and neurodegenerative diseases. Reliable quantification of WMHs is essential for understanding their clinical impact in normal and pathological populations. Automated segmentation of WMHs is highly challenging due to heterogeneity in WMH characteristics between deep and periventricular white matter, presence of artefacts and differences in the pathology and demographics of populations. In this work, we propose an ensemble triplanar network that combines the predictions from three different planes of brain MR images to provide an accurate WMH segmentation. In the loss functions the network uses anatomical information regarding WMH spatial distribution in loss functions, to improve the efficiency of segmentation and to overcome the contrast variations between deep and periventricular WMHs. We evaluated our method on 5 datasets, of which 3 are part of a publicly available dataset (training data for MICCAI WMH Segmentation Challenge 2017 - MWSC 2017) consisting of subjects from three different cohorts, and we also submitted our method to MWSC 2017 to be evaluated on the unseen test datasets. On evaluating our method separately in deep and periventricular regions, we observed robust and comparable performance in both regions. Our method performed better than most of the existing methods, including FSL BIANCA, and on par with the top ranking deep learning methods of MWSC 2017.

## Highlights

• We propose TrUE-Net for automated white matter hyperintensities segmentation.
• TrUE-Net uses spatial distribution of WMHs in the loss functions during optimisation.
• TrUE-Net provides robust segmentation of both deep and periventricular WMHs.
• Evaluated on 5 datasets and unseen data of MICCAI WMH segmentation challenge (MWSC).
• TrUE-Net performs better than BIANCA and on par with top ranking MWSC methods.

## Graphical abstract

**Figure:**
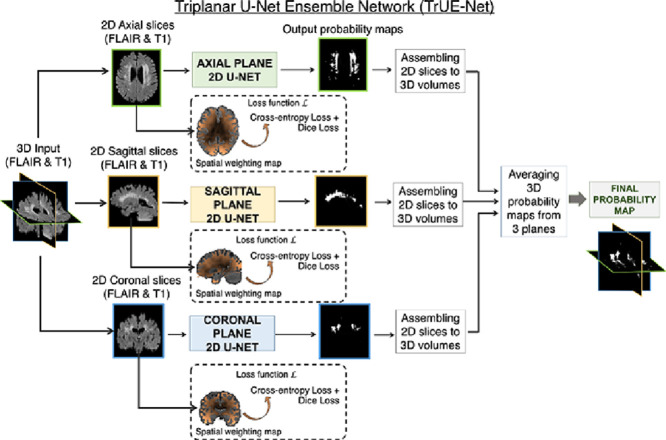

## 1 Introduction

White matter hyperintensities (WMHs) of presumed vascular origin appear as bright localised areas in T2-weighted and fluid-attenuated inversion recovery (FLAIR) images (Wardlaw et al., 2013), and could appear hypointense on T1-weighted images. WMHs occur commonly in patients with cerebrovascular diseases (Li, Simoni, Küker, Schulz, Christie, Wilcock, Rothwell, 2013, Simoni, Li, Paul, Gruter, Schulz, Küker, Rothwell, 2012, Zamboni, Griffanti, Mazzucco, Pendlebury, Rothwell, 2019) and have been associated with cognitive decline, atrophy and neurodegenerative diseases such as dementia (Debette, Beiser, DeCarli, Au, Himali, Kelly-Hayes, Romero, Kase, Wolf, Seshadri, 2010, Prins, Scheltens, 2015, Pantoni, Basile, Pracucci, Asplund, Bogousslavsky, Chabriat, Erkinjuntti, Fazekas, Ferro, Hennerici, et al., 2005). However, they are also found commonly in healthy elderly subjects (Wardlaw et al., 2013). Therefore, the relationship between the occurrence of WMHs and various clinical factors is not yet fully understood. While various visual rating scales are available (Fazekas et al., 1987, Scheltens, Barkhof, Leys, Pruvo, Nauta, Vermersch, Steinling, Valk, 1993, Wahlund, Barkhof, Fazekas, Bronge, Augustin, Sjögren, Wallin, Ader, Leys, Pantoni, et al., 2001) and can provide qualitative or categorical information regarding WMHs, these scales provide limited information regarding their spatial distribution. Voxel-wise WMH maps, on the other hand, enable more precise quantification of WMHs and open up the possibility of studying the relationship between the spatial location/distribution of WMHs and various clinical factors. In turn, this helps to identify patterns of normal and pathological ageing (Rostrup, Gouw, Vrenken, van Straaten, Ropele, Pantoni, Inzitari, Barkhof, Waldemar, Group, et al., 2012, Biesbroek, Kuijf, van der Graaf, Vincken, Postma, Mali, Biessels, Geerlings, Group, et al., 2013). Hence, location and volume-based lesion characterisation are being increasingly considered in clinical setting (Wardlaw, Smith, Biessels, Cordonnier, Fazekas, Frayne, Lindley, T O’Brien, Barkhof, Benavente, et al., 2013, Smith, Biessels, De Guio, de Leeuw, Duchesne, Düring, Frayne, Ikram, Jouvent, MacIntosh, et al., 2019). Given the importance of analysing the clinical impact of WMHs, especially in large cohorts, manual segmentation of WMHs is time consuming and is prone to intra/inter-rater variability. Hence, an automated method to provide exact voxel-level localisation and accurate quantification of WMHs would be highly useful.

Several automated WMH segmentation methods have been proposed (Caligiuri et al., 2015) using features based on intensity (Ong, Ramachandram, Mandava, Shuaib, 2012, Damangir, Manzouri, Oppedal, Carlsson, Firbank, Sonnesyn, Tysnes, O’Brien, Beyer, Westman, et al., 2012), combined with anatomy (Ong, Ramachandram, Mandava, Shuaib, 2012, Damangir, Manzouri, Oppedal, Carlsson, Firbank, Sonnesyn, Tysnes, O’Brien, Beyer, Westman, et al., 2012) and appearance (such as shape, contrast etc.) (Samaille, Fillon, Cuingnet, Jouvent, Chabriat, Dormont, Colliot, Chupin, 2012, Griffanti, Zamboni, Khan, Li, Bonifacio, Sundaresan, Schulz, Kuker, Battaglini, Rothwell, et al., 2016). Among the existing methods using hand-crafted features, unsupervised methods such as clustering (Admiraal-Behloul, Van Den Heuvel, Olofsen, van Osch, van der Grond, Van Buchem, Reiber, 2005, Ong, Ramachandram, Mandava, Shuaib, 2012, Samaille, Fillon, Cuingnet, Jouvent, Chabriat, Dormont, Colliot, Chupin, 2012), supervised classification algorithms (Anbeek, Vincken, Van Osch, Bisschops, Van Der Grond, 2004, Damangir, Manzouri, Oppedal, Carlsson, Firbank, Sonnesyn, Tysnes, O’Brien, Beyer, Westman, et al., 2012, Yoo, Lee, Han, Lee, MacFall, Payne, Kim, Kim, Kim, et al., 2014, Griffanti, Zamboni, Khan, Li, Bonifacio, Sundaresan, Schulz, Kuker, Battaglini, Rothwell, et al., 2016) and probabilistic approaches (Yang et al., 2010) have been proposed. Despite the large amount of methods developed for WMH segmentation, only a few of them are publicly available (Damangir, Manzouri, Oppedal, Carlsson, Firbank, Sonnesyn, Tysnes, O’Brien, Beyer, Westman, et al., 2012, Lao, Shen, Liu, Jawad, Melhem, Launer, Bryan, Davatzikos, 2008, Schmidt, Gaser, Arsic, Buck, Förschler, Berthele, Hoshi, Ilg, Schmid, Zimmer, et al., 2012, Griffanti, Zamboni, Khan, Li, Bonifacio, Sundaresan, Schulz, Kuker, Battaglini, Rothwell, et al., 2016). Using hand-crafted features might not be sufficient to capture the lesion patterns and to overcome noise and artefacts. The lesion characteristics of WMHs show high variability depending on their location, making their segmentation challenging. For instance, between periventricular WMHs (PWMHs) and deep WMHs (DWMHs) (Griffanti et al., 2017), PWMHs usually appear brighter, larger and often form confluent lesions, with higher contrast when compared to DWMHs that usually occur as small punctate lesions. Also, the lesion load and distribution are influenced by demographic factors (e.g. age) and clinical conditions (e.g. hypertension). Additionally, artefacts (e.g. Gibbs ringing, motion artefacts) and noise that occur during image acquisition also affect the segmentation performance. Also, most of the methods have been evaluated on a limited number of subjects (Anbeek, Vincken, Van Osch, Bisschops, Van Der Grond, 2004, De Boer, Vrooman, Van Der Lijn, Vernooij, Ikram, Van Der Lugt, Breteler, Niessen, 2009, Yang, Shan, Kruggel, 2010, Steenwijk, Pouwels, Daams, van Dalen, Caan, Richard, Barkhof, Vrenken, 2013, Khademi, Venetsanopoulos, Moody, 2011, Yoo, Lee, Han, Lee, MacFall, Payne, Kim, Kim, Kim, et al., 2014) or on a specific population with limited demographic and pathological characteristics (Gibson, Gao, Black, Lobaugh, 2010, Jeon, Yoon, Park, Seo, Kim, Kim, Kim, Na, Lee, 2011).

Deep learning (DL) allows computational models with multiple layers to learn data representations at different layers of abstraction (LeCun et al., 2015), thus utilising more contextual information compared to the hand-crafted features. With increase in computational resources, such as graphical processing units (GPUs) and techniques such as data augmentation (Russakovsky et al., 2015), DL has been quickly emerging as a reliable segmentation tool in biomedical imaging, especially for lesion segmentation (Guerrero, Qin, Oktay, Bowles, Chen, Joules, Wolz, Valdés-Hernández, Dickie, Wardlaw, et al., 2018, Ghafoorian, Karssemeijer, Heskes, van Uden, Sanchez, Litjens, de Leeuw, van Ginneken, Marchiori, Platel, 2017). For instance, in the MICCAI WMH Segmentation Challenge 2017 (MWSC 2017, https://wmh.isi.uu.nl, Kuijf et al. (2019)), out of 20 methods competing in the initial call, 14 of them (including the top ranking methods) were based on DL. Existing DL methods for WMH segmentation have used convolutional neural network (CNN) models, including various ensemble models (Li, Jiang, Zhang, Wang, Wang, Zheng, Menze, 2018, Kuijf, Biesbroek, de Bresser, Heinen, Andermatt, Bento, Berseth, Belyaev, Cardoso, Casamitjana, et al., 2019), encoder-decoder models (Li, Simoni, Küker, Schulz, Christie, Wilcock, Rothwell, 2013, Guerrero, Qin, Oktay, Bowles, Chen, Joules, Wolz, Valdés-Hernández, Dickie, Wardlaw, et al., 2018, Kuijf, Biesbroek, de Bresser, Heinen, Andermatt, Bento, Berseth, Belyaev, Cardoso, Casamitjana, et al., 2019, Zhang, Chen, Chen, Tang, 2018) - especially U-Nets (proposed by Ronneberger et al. (2015)), 3D multi-dimensional gated recurrent networks (Andermatt et al., 2016) and ResNets (Guerrero et al., 2018). The common choices of inputs used for these networks have been 2D slices (Li et al., 2018) or small 2D/3D patches at multiple scales (Ghafoorian, Karssemeijer, Heskes, van Uden, Sanchez, Litjens, de Leeuw, van Ginneken, Marchiori, Platel, 2017, Andermatt, Pezold, Cattin, 2016). In general, the choice of the model dimension is affected by various factors, such as size and distribution of the lesions and the amount of data available. Various architectures of 3D CNN models have been successfully used in the segmentation of various types of larger lesions (Kamnitsas, Ledig, Newcombe, Simpson, Kane, Menon, Rueckert, Glocker, 2017, Havaei, Davy, Warde-Farley, Biard, Courville, Bengio, Pal, Jodoin, Larochelle, 2017, Oktay, Schlemper, Folgoc, Lee, Heinrich, Misawa, Mori, McDonagh, Hammerla, Kainz, et al., 2018). However, images with WMHs include small lesions in the deep regions, with low contrast and poor context, often with poor resolution along the *z*-dimension. This constrains the accurate detection of WMH boundary voxels using 3D CNNs (Li et al., 2018). From the implementation point of view, 2D models use far fewer parameters when compared to 3D models. However, using 2D models can cause discontinuities in segmentation across the *z*-dimension since individual slices are considered separately. Therefore, to leverage the advantages of both 2D and 3D models, methods utilizing a 2.5D architecture, or with 2D models applied individually on each of the 3 planes of the image, have been proposed for various segmentation applications (Aslani, Dayan, Storelli, Filippi, Murino, Rocca, Sona, 2019, Piantadosi, Sansone, Fusco, Sansone, 2020, Prasoon, Petersen, Igel, Lauze, Dam, Nielsen, 2013, Roth et al., 2014). These triplanar approaches have shown to avoid parameters explosion (Piantadosi et al., 2020), as in the case of 3D models, and provide better segmentation and continuity across slices than the 2D models. Another important aspect that changes widely between different datasets and can impact the model performance is voxel resolution. Most of the DL methods (including 3D and 2D methods) have been evaluated on images with isotropic voxels, mainly with axial acquisitions (Ghafoorian, Karssemeijer, Heskes, van Uden, Sanchez, Litjens, de Leeuw, van Ginneken, Marchiori, Platel, 2017, Kuijf, Biesbroek, de Bresser, Heinen, Andermatt, Bento, Berseth, Belyaev, Cardoso, Casamitjana, et al., 2019), while acquisition protocol variations (with anisotropic voxels and/or different axis of acquisition) are common in the real-world clinical datasets. Regarding the method accessibility, in the case of DL methods, not many are publicly available. In fact, even if most of the pre-trained models from MWSC 2017 challenge are publicly available for testing, there are no independent DL tools available that allow users to train/fine-tune the models on their own data for improving segmentation performance on various datasets from different scanners/centres.

We aimed to develop a DL tool that provides highly accurate segmentation in both periventricular and deep regions, that is publicly available, and that has the flexibility to change training hyperparameters options on various datasets. In this work, we propose TrUE-Net (Triplanar U-Net ensemble network), a DL method for segmentation of WMHs, consisting of an ensemble of U-Nets, each applied to one of the three planes (axial, sagittal and coronal) planes of structural brain MR images. We aim to improve WMH segmentation irrespective of lesion location and lesion load by training the TrUE-Net using loss functions that take into account the anatomical location and distribution of WMHs. We evaluate the proposed model on 5 different datasets with different acquisition (scanner and MRI protocol) and lesion characteristics: one from a study on neurodegeneration in prodromal and manifest Alzheimer’s disease, one from a vascular cohort (coronal images with anisotropic voxels), and three from a publicly available training dataset from MWSC 2017. Additionally, our method was also evaluated on an unseen test data in MWSC 2017, which includes, in addition to data from the same centres as that of the publicly available MWSC training data, data acquired using two different scanners (a 1.5T and a PET-MR system). We compared the performance of TrUE-Net against methods using hand-crafted features and DL methods, with respect to manual segmentations. Initially, we performed a direct comparison between the results of TrUE-Net and BIANCA, the existing FSL (FMRIB software library) WMH segmentation tool (Griffanti et al., 2016). Later, we compared the TrUE-Net results with those of the top performing method (Li et al., 2018) from the MWSC 2017 on various datasets. Finally, we performed indirect comparisons of TrUE-Net results against various existing methods.

## 2 Materials and methods

### 2.1 Triplanar U-Net Ensemble Network (TrUE-Net)

#### 2.1.1 Preprocessing

We used both T1-weighted and FLAIR images as inputs for the model. We reoriented the images to the standard MNI space, performed skull-stripping with FSL BET (Smith, 2002) and bias field correction using FSL FAST (Zhang et al., 2001). We registered the T1-weighted image to the FLAIR using linear rigid-body registration (Jenkinson and Smith, 2001) and cropped the field of vision (FOV) close to the brain and applied Gaussian normalisation to normalise the intensity values. We then extracted 2D slices from the volumes from all three planes. For the axial plane, we cropped the slices to a dimension of 128 × 192 voxels. For sagittal and coronal slices, we cropped and resized the extracted slices to 192 × 120 and 128 × 80 voxels respectively, using bilinear interpolation.

#### 2.1.2 TrUE-Net architecture

The proposed triplanar architecture consists of three 2D networks, each one detecting WMHs from a different plane. The triplanar network reduces discontinuities in WMH segmentation across slices and provides better and comprehensive lesion boundary delineation, using fewer parameters compared to a 3D CNN.

In TrUE-Net, we combined three 2D U-Nets in parallel within an ensemble model. In the ensemble architecture, variation in the individual probability maps (due to noise or spurious structure) is reduced when they are combined in the ensemble network.

**Fig. 1:**
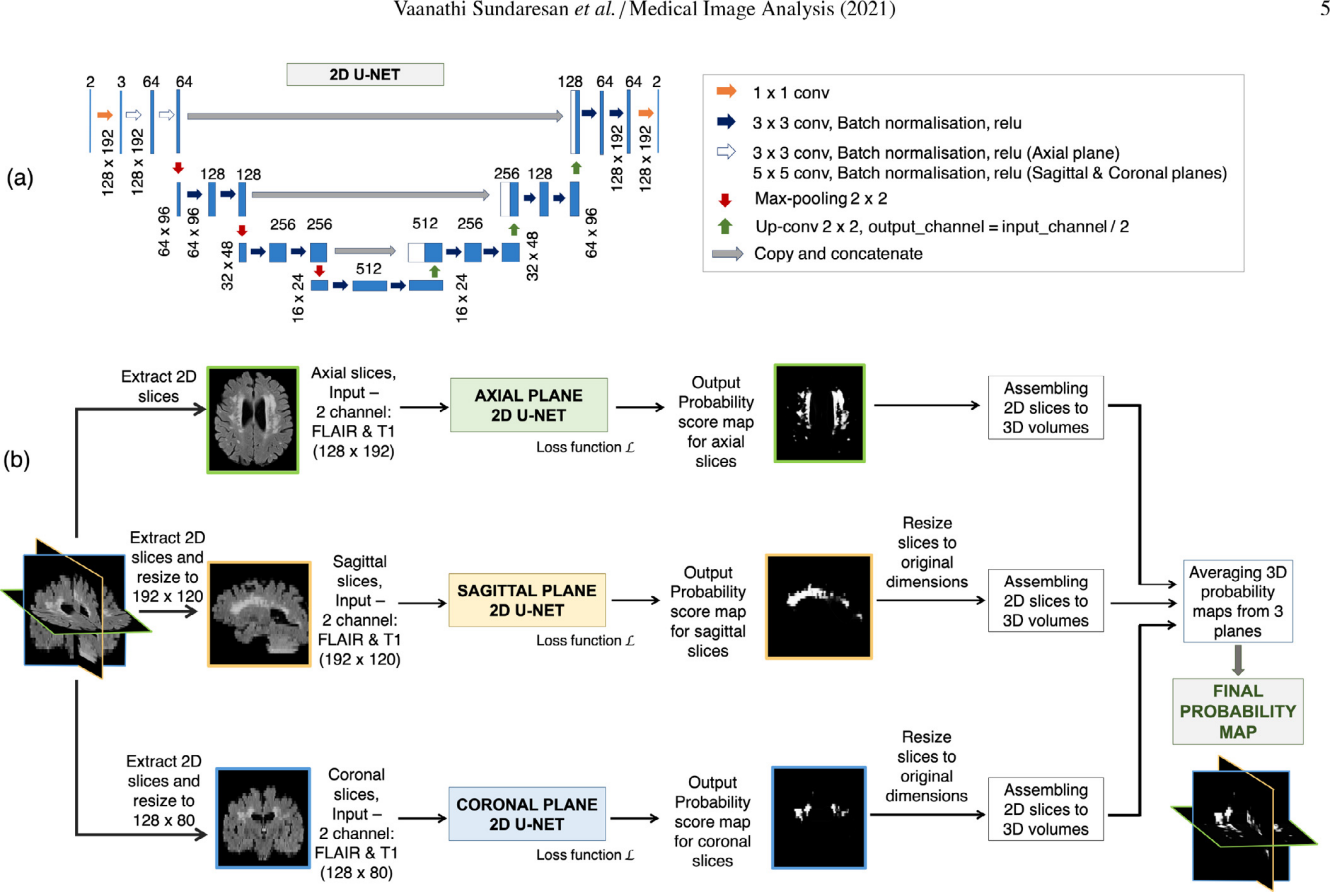
Triplanar U-Net ensemble network (TrUE-Net). (a) U-Net model used in individual planes, (b) Overall TrUE-Net architecture.

Fig. 1 shows the architecture of the proposed TrUE-Net. For each plane, the 2D model takes FLAIR and T1-weighted slices as input channels and provides the probability map in the corresponding plane. In each plane, we trimmed the depth of the classic U-Net (Ronneberger et al., 2015) to obtain a 3-layer deep U-Net model. This reduces the computational load and improves the model sensitivity towards small lesions. Our model mostly uses 3 × 3 convolutional kernels, except for the initial 5 × 5 convolutional kernels in the first layer of the sagittal and coronal U-Nets (Fig. 1(a)), since larger receptive fields could aid in learning more generic lesion patterns in these planes, thus reducing discontinuities across slices. Each convolution layer is followed by a batch normalisation layer and an activation layer (using *ReLU* - rectified linear unit). We added a 1 × 1 convolutional kernel at the end, before the softmax layer for predicting the probability maps. In the ensemble model, training of U-Nets in the individual planes occurs independently, using the slices extracted from the corresponding planes from the resized training images. During testing, for each network we assembled the slices into a 3D probability map. We then resized each 3D map back to the original dimension and finally averaged the three 3D maps to obtain the final probability map.

#### 2.1.3 Loss function

**Fig. 2:**
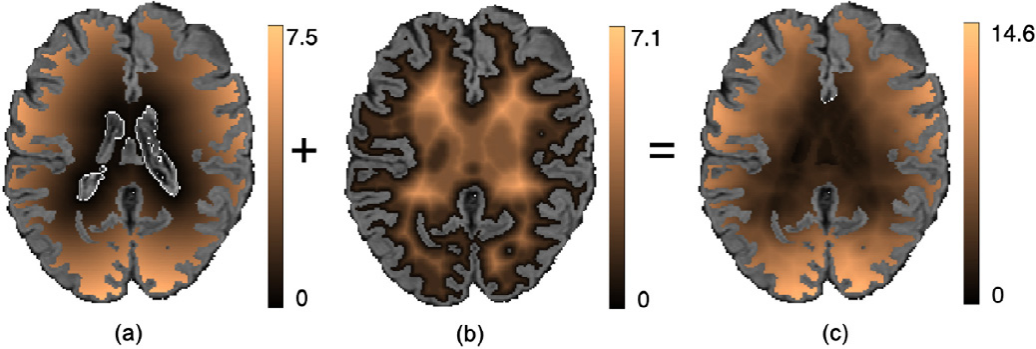
Example weight maps for weighting the voxel-wise cross-entropy loss function. Maps showing the sum of (a) Distance from ventricles $D_{vent}$ and (b) Distance from gray matter (GM) $D_{GM}$ to get (c) Final weight map $D_{wei}$. The spatial weights (distances) are calculated for the whole brain. Here, the weight maps are shown after applying the WM mask from the post-processing step (see Section 2.1.4) for illustration purpose..

We used a weighted sum of the voxel-wise cross-entropy (CE) loss function and the Dice loss (DcL) as the total cost function. The CE loss aims to make the segmentation better at the image-level and is biased towards the detection of larger periventricular WMHs. Hence, we weighted the CE loss function using a spatial weight map (an example shown in Fig. 2) to up-weight the deep areas that have high class imbalance (i.e. contain fewer WMHs compared to the background). The addition of the Dice loss also helps with deep WMHs, since missing small WMHs would make more difference to the Dice component than to the CE loss component. Hence, training the network with the inclusion of a Dice component in the loss function would favour finding small lesions and reducing false negatives, especially in the areas of high class imbalance (Milletari, Navab, Ahmadi, 2016, Li, Jiang, Zhang, Wang, Wang, Zheng, Menze, 2018).

For each subject, we determined the distance from ventricles using the FSL distancemap command, $0\leq D_{vent}\leq N_{D}$ (Fig. 2a), where $N_{D}$ is the maximum distance from ventricles to the boundary of the brain mask, and the distance from the brain gray matter (GM, derived from the cortical CSF segmentation obtained with FSL FAST^2^), $0\leq D_{GM}\leq M_{D}$ (Fig. 2b), where $M_{D}$ is the maximum distance from the GM boundary to the centre of the brain. We then obtained the final weight map as the sum of both, $D_{wei}=D_{vent}+D_{GM},0\leq D_{wei}\leq \left(N_{D}+M_{D}\right)$ (Fig. 2c) (all distances in mm). The sum of these two distance values ensures that the deep region receives higher weights than the periventricular region, and simultaneously avoids relying on GM segmentation that could contain misclassified WMH voxels. Hence the total weighted CE loss for N voxels is given by,

(1) $$CE=-\sum_{x=1}^{N}\sum_{i=1}^{C}\;y\left(x\right)\;D_{wei}\left(x\right)\;\text{log}\left(p\left(x\right)\right)$$

where p(x) denotes the output of the soft-max layer, C is the number of classes and y(x) = 0 or 1, the value at each voxel x on the manual segmentation. Given the manual segmentation M and binary map $P_{th}$ obtained by thresholding predicted probability maps, the Dice loss for N voxels is given by,

(2) $$DcL=-\frac{2\times \sum_{x=1}^{N}M\left(x\right)\cdot P_{th}\left(x\right)}{\sum_{x=1}^{N}M\left(x\right)+\sum_{x=1}^{N}P_{th}\left(x\right)}$$

Hence the total loss function L is given by,

(3) L=CE+DcL

(4) $$\begin{array}{cc} = & -\sum_{x=1}^{N}\sum_{i=1}^{C}y\left(x\right)\;D_{wei}\left(x\right)\;\text{log}\left(p\left(x\right)\right)\\ & -\frac{2\times \sum_{x=1}^{N}M\left(x\right)\cdot P_{th}\left(x\right)}{\sum_{x=1}^{N}M\left(x\right)+\sum_{x=1}^{N}P_{th}\left(x\right)} \end{array}$$

We chose equal weights on the basis of initial empirical experiments (not shown) for the Dice loss and the weighted CE loss while adding them to obtain the total loss. The range of CE loss function is ideally 0 ≤ CE loss ≤ W (where W is determined from the spatial weight map) and the range of Dice loss is -1 ≤ Dice loss ≤ 0. Therefore, the range of the sum of these two loss values is -1 ≤ (CE loss + Dice loss) ≤ W. Although the total loss function can assume negative values, in practice, the CE loss hardly converged at the value close to 0 and applying weight values (W) resulted in CE loss values > 0 at convergence. Therefore, even with the addition of Dice loss, the total loss value converged around 0 (or small negative values, e.g. ≥ -0.1). However, it must be noted that during optimisation, it is not the value of the loss that is important but the change in loss across parameter space, since the weights of the model are updated using the gradient of the loss and not the loss value itself.

#### 2.1.4 Post-processing

We padded the output probability maps with zeros to bring them back to their original dimensions. We later masked the probability map with the white matter mask obtained from a dilated and inverted cortical CSF tissue segmentation (using FSL FAST (Zhang et al., 2001)) combined with other deep grey exclusion masks (make_bianca_mask command in FSL BIANCA (Griffanti et al., 2016)). Finally, we thresholded the masked probability maps at 0.5.

#### 2.1.5 Implementation details

We implemented the network in Python 3.6 using Pytorch 1.2.0. The network was trained on an NVIDIA Tesla V100, taking 45 seconds (for 3 planes) per epoch for 15,000 samples with the training/validation split of 90%/10%. For each leave-one-out (LOO) evaluation, we excluded the test subject and randomly sampled 10% of the remaining subjects as the validation dataset while using the 90% for training the model. We used a patience (number of epochs to wait for progress on validation set) value of 20 to determine the convergence (early stopping). The model converged at around 80 epochs for all the datasets. We used the Adam Optimiser with $\epsilon \;=\;10^{-4}$. We used a batch size of 8, with an initial learning rate of $1\times 10^{-3}$ and reducing it by a factor $1\times 10^{-1}$ every 2 epochs, until it reaches $1\times 10^{-5}$, after which we maintain the fixed learning rate value. We chose the above parameters empirically based on the model convergence (refer to Section 2.3.2 for more details). Data augmentation was applied using translation (x/y-offset ∈ [-10, 10]), rotation (θ ∈ [-10, 10]), random noise injection (Gaussian, μ = 0, $\sigma^{2}$ ∈ [0.01, 0.09]) and Gaussian filtering (σ ∈ [0.1, 0.3]), increasing the dataset by a factor of 10 and 6 for axial and sagittal/coronal planes respectively. The hyperparameter values for the data augmentation transformations were randomly sampled from the closed intervals specified above using a uniform distribution.

### 2.2 Datasets

#### 2.2.1 Neurodegenerative cohort (NDGEN)

The dataset, used in Zamboni et al. (2013), includes MRI data from 9 subjects with probable Alzheimer’s Disease, 5 with amnestic mild cognitive impairment and 7 cognitively healthy control subjects (age range 63 - 86 years; mean age 77.1 ± 5.8 years; median age 77 years; F:M = 10:11). Total brain volume range: 1,189,282 - 1,614,799 mm${}^{3}$, median: 1,424,669 mm${}^{3}$. Manual segmentation was available for all datasets (WMH load range: 1,878 - 89,259 mm${}^{3}$, median: 20,772 mm${}^{3}$). The images were acquired using a 3T Siemens Trio Scanner, with FLAIR (TR/TE = 9,000/89 ms, flip angle 150${}^{o}$, FOV 220 mm, voxel size 1.1 × 0.9 × 3 mm, matrix size 256 × 256 × 35 voxels) and T1-weighted sequence (3D MP-RAGE sequence, TR/TE = 2,040/4.7 ms, flip angle 8${}^{o}$, FOV 192 mm, voxel size 1 mm isotropic, matrix size 174 × 192 × 192 voxels).

#### 2.2.2 Vascular cohort - Oxford vascular study (OXVASC)

The dataset consists of 18 participants in the OXVASC study (Rothwell et al., 2004), who had recently experienced a minor non-disabling stroke or transient ischemic attack (age range 50 - 91 years; mean age 73.27 ± 12.32 years; median age 75.5 years; F:M = 7:11). Total brain volume range: 1,290,926 - 1,918,604 mm^3^, median: 1568233 mm^3^. Manual segmentation was available for all datasets (WMH load range: 3,530 - 83,391 mm^3^, median: 16,906 mm^3^). The images were acquired using a 3T Siemens Trio Scanner, with FLAIR (TR/TE = 9,000/88 ms, flip angle 150${}^{o}$, voxel size 1 × 3 × 1 mm, matrix size 174 × 52 × 192 voxels), T1-weighted sequence (3D MP-RAGE sequence, TR/TE = 2,000/1.94 ms, flip angle 8${}^{o}$, voxel size 1 mm isotropic, matrix size 208 × 256 × 256 voxels) and diffusion-weighted imaging (TR/TE = 8,000/86 ms, GRAPPA factor 2, flip angle 16${}^{o}$, FOV 192 mm, voxel size 2 × 2 × 2 mm, 32 directions, b value 1,500 s/mm${}^{2}$).

#### 2.2.3 MICCAI WMH Segmentation challenge training dataset (MWSC)

The dataset consists of 60 subjects from three different sources (20 subjects each) provided as training sets for the challenge (http://wmh.isi.uu.nl/): UMC Utrecht, NUHS Singapore and VU Amsterdam. The brain volume ranges: 1,257,820 - 1,844,920 mm^3^ (median 1,473,389 mm^3^) for UMC Utrecht, 1,147,248 - 1,532,268 mm^3^ (median: 1,351,325 mm^3^) for NUHS Singapore and 1,219,614 - 1,787,321 mm^3^ (median: 1,441,201 mm^3^) for VU Amsterdam. Manual segmentations were available for all three datasets, with an additional exclusion label provided for other pathologies. In the challenge, the masks with exclusion labels were ignored during performance evaluation. However, we included these masks as parts of non-lesion tissue, during both training and testing, for the calculation of the performance metrics in order to get more stringent evaluation in the presence of other pathologies. The WMH volume ranges (excluding other pathologies) are 845 - 74,991 mm^3^ (median: 26,240 mm^3^) for UMC Utrecht, 786 - 61,332 mm^3^ (median: 17,795 mm^3^) for NUHS Singapore and 1,522 - 43,528 mm^3^ (median: 6,015 mm^3^) for VU Amsterdam. For more details regarding MRI acquisition parameters, refer to http://wmh.isi.uu.nl/.

#### 2.2.4 MWSC Test data

This is an in-house dataset used only for evaluation by the organisers, consisting of 110 subjects from five different sources. Out of the total number of subjects, 90 subjects were from the three sources (UMC Utrecht, NUHS Singapore and VU Amsterdam), 30 subjects from each source, mentioned above for the training data. The remaining 20 images were from VU Amsterdam using different scanners - 3T Philips Ingenuity and 1.5T GE Signa HDxt, with 10 subjects from each scanner. For more details regarding MRI acquisition parameters, refer to http://wmh.isi.uu.nl/.

We used the NDGEN dataset for the initial model optimisation, to observe the effect of hyperparameters, model dimension and loss function components (sections 2.3.2–2.3.4). We then evaluated the optimised model on the NDGEN, OXVASC and MWSC datasets using leave-one-out evaluation (Section 2.3.5) and compared it with exiting methods (2.3.6–2.3.7). Finally, we performed external validation of our method on the MWSC 2017 unseen test dataset (Section 2.3.5).

### 2.3 Experiments

#### 2.3.1 Performance evaluation metrics

We used the following performance metrics in our evaluation:

• **Dice Similarity Index (SI)**, calculated as 2 × (true positive WMH voxels) / (true WMH voxels + positive WMH voxels).
• **Voxel-wise true positive rate (TPR)** is the ratio of the number of true positive WMH voxels to the number of true WMH voxels.
• **Voxel-wise false positive rate (FPR)** is the number of false positive (FP) WMH voxels divided by the number of non-WMH voxels.
• **Cluster-wise TPR** is the number of true positive WMH clusters divided by the total number of true WMH clusters.
• **Absolute volume difference (AVD) (%)** is the absolute difference between the volume of manually segmented WMHs voxels and the volume of detected WMHs voxels, as a percentage of the manually segmented lesion voxels.
• **Cluster-wise F1-measure** = $\frac{2\times (Cluster-wise\;TPR\times Cluster-wise\;precision)}{\left(Cluster-wise\;TPR+Cluster-wise\;precision\right)}$,
  where Cluster-wise precision is the number of true positive WMH clusters divided by the total number of detected WMH clusters.
• **95**^th^**percentile of Hausdorff distance measure (H95)**: We determined the set of the closest distances between the points on the detected WMH boundary and the manually segmented WMH boundary. We calculated the 95^th^ percentile of this set of closest distance values, instead of the maximum value used for the standard Hausdorff value (since the former is less prone to noise).

It is important to note that there is some degree of uncertainty associated with the delineation of boundaries of diffuse lesions in both manual segmentations and the predicted outputs, which could affect voxel-wise evaluation metrics and H95. Hence, we also used various cluster-wise metrics for evaluating the segmentation performance. In cluster-wise metrics we adopted a 26-connected neighbourhood to define clusters.

#### 2.3.2 Effect of training hyperparameters on model performance

We used the NDGEN dataset for the initial optimisation of network parameters, and for determining the number of epochs. We explored the effect of batch-size, learning rate and epsilon (ϵ) value of the Adam optimiser on model convergence. We experimented with three batch sizes: 8, 16 and 32, and three ϵ values: $1\times 10^{-2}$, $1\times 10^{-4}$ and $1\times 10^{-6}$. For learning rate, we tested the effect of following 3 settings: (i) **Higher:** Initially $1\times 10^{-2}$, reducing by a factor $1\times 10^{-1}$ every 2 epochs until it reaches $1\times 10^{-4}$ and keeping it constant afterwards, (ii) **Medium:** Initially $1\times 10^{-3}$, reducing by a factor $1\times 10^{-1}$ every 2 epochs until it reaches $1\times 10^{-5}$ and (iii) **Lower:** Initially $1\times 10^{-4}$, reducing by a factor $1\times 10^{-1}$ every 2 epochs until it reaches $1\times 10^{-6}$.

#### 2.3.3 Ablation study: Effect of loss function components

We performed an ablation study to determine the effect of the components of the loss function on the segmentation results using the NDGEN dataset. For this experiment, we removed one component of the loss function at a time and compared the performance of TrUE-Net with three cases of loss functions: cross-entropy loss, weighted cross-entropy loss and weighted cross-entropy loss + Dice loss. We also compared the weighted cross-entropy loss + Dice loss with the case of using only the Dice loss component. Since we hypothesized that the weighting of the CE loss would specifically improve the performance in deep regions, we determined SI values in periventricular and deep regions separately, and compared the results between regions. Due to the small sample size and the non-Gaussian distribution of the data in most cases (Shapiro-Wilks test), we performed non-parametric statistics using Wilcoxon signed rank test. We adopted the 10 mm distance rule (DeCarli, Fletcher, Ramey, Harvey, Jagust, 2005, Griffanti, Jenkinson, Suri, Zsoldos, Mahmood, Filippini, Sexton, Topiwala, Allan, Kivimäki, et al., 2018) for classification of PWMH and DWMH.

#### 2.3.4 Effect of model dimension

We also determined the effect of the dimension of the model on the segmentation performance using the NDGEN dataset. We removed one aspect of model dimension at a time and compared three cases: 3D U-Net, 2D U-Net (on axial plane only) and TrUE-Net. Similar to the above study, we determined SI values in periventricular and deep regions separately and performed Wilcoxon signed rank test to compare between regions.

We maintained the same training and model parameters for all three options in both studies.

#### 2.3.5 Evaluation of WMH segmentation

After optimising the proposed model, we performed leave-one-out (LOO) evaluation of our proposed model separately on the following datasets: (i) MWSC (combined from Utrecht, Singapore and Amsterdam cohorts), (ii) NDGEN and (iii) OXVASC, based on the performance metrics specified in Section 2.3.1. We chose to perform LOO evaluation rather than fold validation since the former provides more unbiased performance estimates (as it uses a larger training data (Elisseeff et al., 2003)) and provides more reliable estimates in smaller datasets (e.g. NDGEN and OXVASC). For comparison, the results of 3-fold cross validation on the MWSC dataset are reported in the supplementary material. Also, we submitted a docker container of our method (trained on the MWSC dataset) to the MWSC 2017 for evaluating our method on the unseen MWSC test data. The submitted container was validated on the test datasets by the organisers and the results were provided for the individual test datasets, along with the weighted average of performance metrics (weighted by the number of subjects in the dataset). On the MWSC, NDGEN and OXVASC datasets, we determined the performance of the model in the deep and the periventricular regions (using 10 mm distance rule as above) separately, and performed paired t-tests to compare the results between regions.

#### 2.3.6 Comparison with BIANCA

We performed a direct comparison of our TrUE-Net with BIANCA using LOO evaluation on the MWSC, NDGEN and OXVASC datasets, based on the performance metrics specified in Section 2.3.1 and performed Wilcoxon signed rank test between TrUE-Net and BIANCA results.

**BIANCA features and training options:** For NDGEN, we used FLAIR and T1 as features and the options that provided the best results in the initial validation of BIANCA (Griffanti et al., 2016). Other than the default options, we used the following non-default options: location of training points = no border, number of training points = Fixed + unbalanced with 2,000 lesion points and 10,000 non-lesion points. The ‘no border’ option and fixed + unbalanced lesion and non-lesion points have been shown to provide the best results during initial validation of BIANCA (Griffanti et al., 2016) and also in our initial tests on the same data. For OXVASC we used FLAIR + T1 + mean diffusivity (MD) as features (refer to Zamboni et al. (2019) for the MD preprocessing details). Specific options used were: sw = 2, 3D patch with patch size of 3. Due to the anisotropic nature of the voxels, additional intensity features obtained by averaging over a smaller 3D patch provided better results during the initial tests. For the MWSC dataset, we trained BIANCA using the same features and BIANCA options used for NDGEN. For all the datasets, we applied a global threshold value of 0.9 on the BIANCA lesion probability maps and masked with the white matter mask (obtained in Section 2.1.4) to obtain the final binary lesion maps.

#### 2.3.7 Comparison with the top-ranking method of MWSC 2017

On the MWSC dataset, we compared the LOO results of TrUE-Net with the subject-wise LOO performance metrics reported in the supplementary table S1 in Li et al. (2018), which is the top-ranking method in MWSC 2017 (Kuijf et al., 2019). Additionally, we performed LOO evaluation of the model proposed in Li et al. (2018)^3^, by training and evaluating their model separately on the OXVASC and NDGEN datasets for the comparison with TrUE-Net. For these experiments, we used the same training hyperparameters as specified in Li et al. (2018). We also performed Wilcoxon signed rank test between the performance measure of TrUE-Net and Li et al. (2018).

#### 2.3.8 Comparison with other existing methods

Finally, we performed an indirect comparison of our results with those obtained by other WMH segmentation methods in the literature (until 2019). We included in our comparisons only methods that achieved minimum Dice overlap metric or voxel-wise TPR of 0.70.

## 3 Results

### 3.1 Effect of training parameters on model performance

**Fig. 3:**
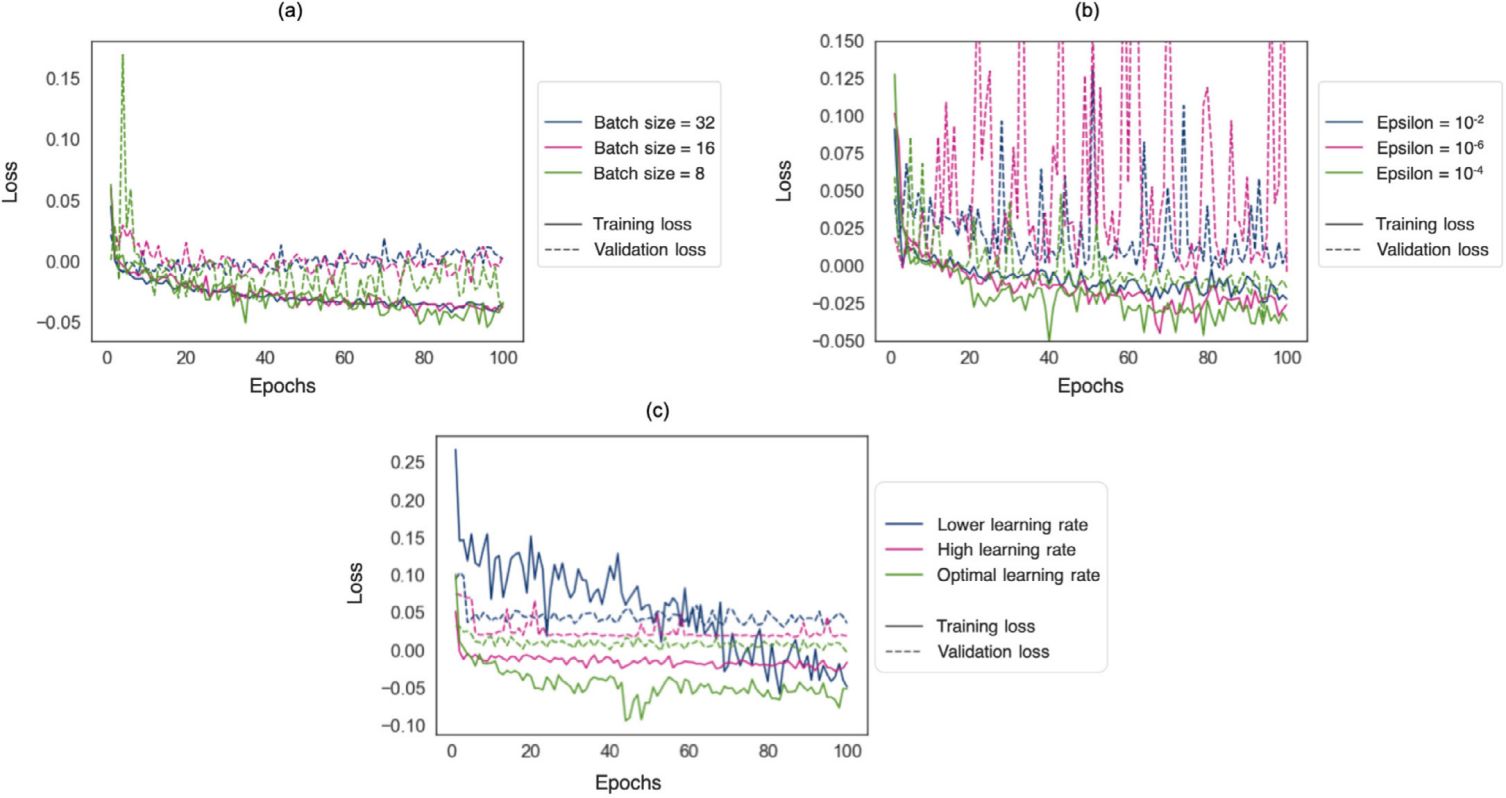
Effect of training parameters on model convergence on the NDGEN dataset. Training and validation loss decays have been shown for different (a) batch sizes, (b) epsilon (ϵ) values and (c) learning rates. The plots shown in green correspond to the optimal values chosen for each parameter. (For interpretation of the references to colour in this figure legend, the reader is referred to the web version of this article.)

Fig. 3 shows the loss function decay with different batch sizes, learning rates and epsilon (ϵ) values. In all the cases, the model started to converge at approximately 80 epochs.

At a batch size of 8, the loss function became noisier, but had lower values. Larger batch sizes resulted in over-fitting, as evident from higher validation loss values for batch sizes of 16 and 32 (Fig. 3a). Therefore, we chose a batch size of 8 for our experiments henceforth. As the ϵ value gets smaller in the denominator, the optimiser makes larger weight updates leading to unstable optimisation, as shown in Fig. 3b for an ϵ value of 1 $\times 10^{-6}$. Since an ϵ value of 1 $\times 10^{-2}$ provided higher loss values (with slightly unstable validation loss), we set the ϵ value to the optimal value of 1 $\times 10^{-4}$ for all the subsequent experiments. From Fig. 3c, for a lower learning rate, the loss decay was slower and hence required more epochs for convergence. On the other hand, for a higher learning rate the loss values values converged before 20 epochs, although with higher loss values. At the optimal learning, the loss decay was slow and converged to a much lower loss value for both training and validation datasets. Hence, we chose the optimal learning rate schedule to be from $1\times 10^{-3}$ to $1\times 10^{-5}$.

### 3.2 Ablation study: Effect of loss function components on segmentation performance

**Fig. 4:**
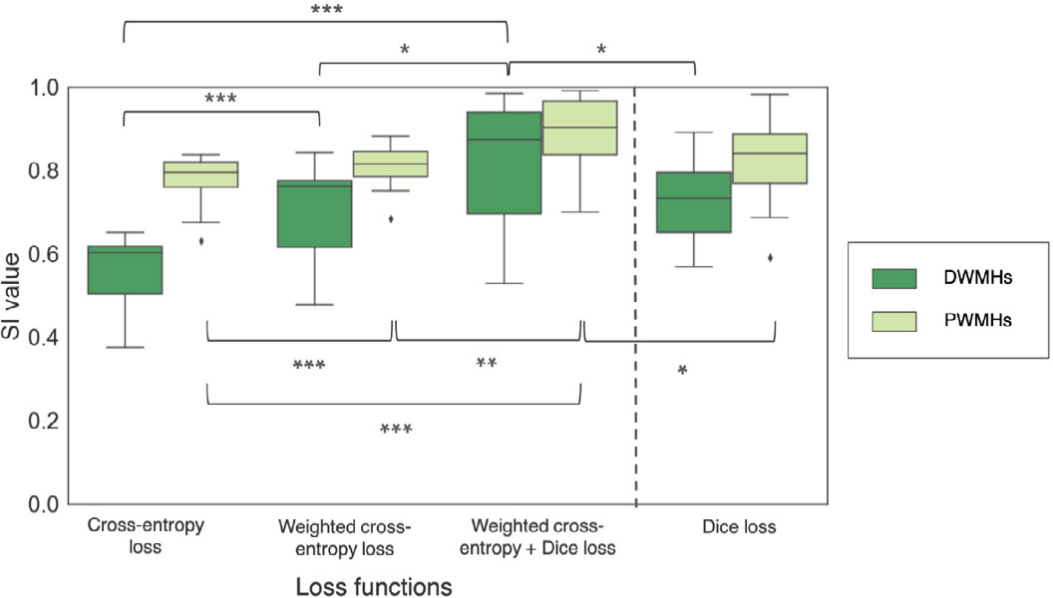
Boxplots of SI values obtained for deep and periventricular regions on the NDGEN dataset for CE, weighted CE and weighted CE + Dice loss functions, compared against the Dice loss function only case. *** - p < 0.0001, ** - p < 0.001, * - p < 0.01.

The boxplots of the SI values for WMH segmentation in deep and periventricular areas are shown in Fig. 4. On performing a non-parametric Friedman test across three components of the loss function (CE loss, weighted CE loss and weighted CE + Dice loss), we found that the SI values significantly increase ($\chi^{2}$ = 25.2, p < 0.0001 for DWMHs, $\chi^{2}$ = 22.9, p < 0.0001 for PWMHs) with the addition of each component of the loss function. With the addition of each component of the loss function, we observed significant improvement in the SI value. Also, weighted CE loss + Dice loss provided significantly higher SI values when compared to CE loss component alone in both regions. As shown in Fig. 4, on applying the Dice loss component alone, we achieved median SI values of 0.73 (with IQR: 0.65 - 0.79) and 0.84 (with IQR: 0.77 - 0.89) for DWMHs and PWMHs respectively, which are significantly lower than those obtained with the combined loss function (p = 0.04 and p = 0.03 for DWMHs and PWMHs respectively).

**Fig. 5:**
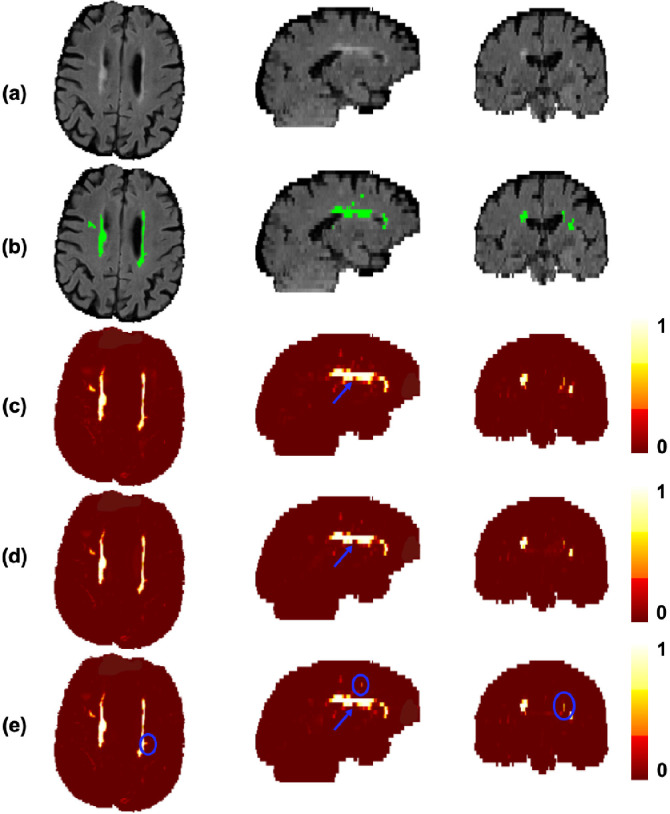
Effect of loss functions on the segmentation performance in the NDGEN dataset. An example showing (a) FLAIR images, (b) manual segmentation against the results of (c) cross-entropy (CE) loss, (d) weighted cross-entropy loss and (e) weighted cross-entropy loss + Dice loss. Differences in lesion segmentations indicated by blue circles and arrows. (For interpretation of the references to colour in this figure legend, the reader is referred to the web version of this article.)

**Fig. 6:**
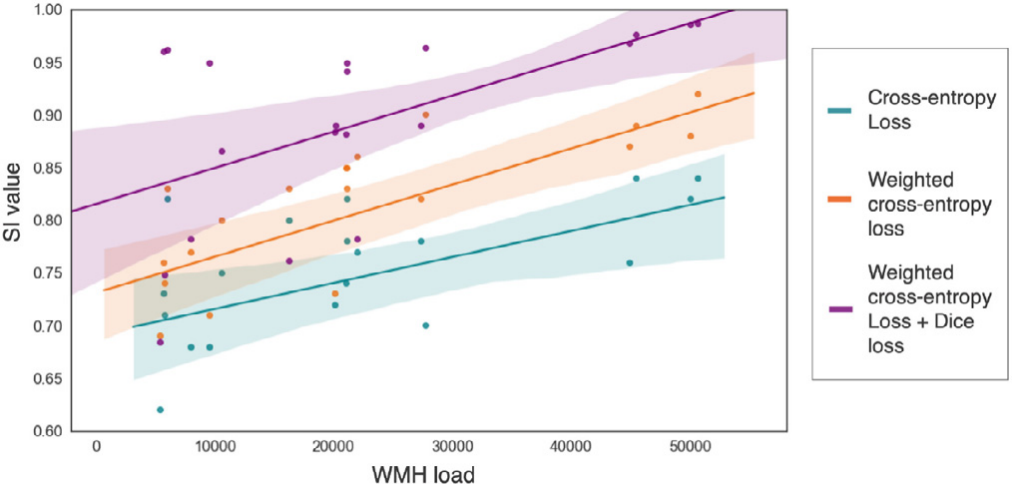
Regression plot showing the impact of lesion load on SI values for weighted cross-entropy loss (magenta) and weighted cross-entropy loss + Dice loss (blue), on the NDGEN dataset. The shaded region represents the 95% confidence interval of the regression estimates. (For interpretation of the references to colour in this figure legend, the reader is referred to the web version of this article.)

Fig. 5 shows the effect of CE and Dice loss components on TrUE-Net segmentation for a subject with medium lesion load. From the boxplots and the visual results, the effect of the composition of the loss function was observable mainly along the edges of PWMHs and DWMHs. In general, we have a large imbalance between WMH and normal WM classes and aim to focus on detecting more true WMHs. Since the CE loss function aggregates the loss values at individual voxels into a global value, it is generally biased towards WMHs in periventricular regions (Fig. 5c) where the imbalance between WMH and non-WMH voxels is less. Therefore, in some cases, the segmentation results extended beyond the ventricle lining. Using weighted CE loss controls this behaviour, since it relies on the weight maps (described in Section 2.1.3) prepared with prior anatomical information. Overcoming this class imbalance, DWMHs are given weights that are more similar to those given to PWMHs. As a result, more DWMHs are detected and the over-segmentation in periventricular regions is avoided (Fig. 5d). Combining the advantages of both the Dice loss and the weighted CE loss components, we obtained better detection of DWMHs, along with accurate segmentation of PWMH borders (Fig. 5). The effect was particularly observable in low lesion load subjects, where the addition of the Dice loss component provided more precise segmentation, with fewer false positives. Fig. 6 shows the effect of lesion load on SI values of TrUE-Net segmentation for the three loss function compositions. In all three cases there was a significant correlation (indicated by Spearman correlation coefficient $\rho_{S}$) between SI and lesion load (CE loss: $\rho_{S}$ = 0.54, p = 0.02; weighted CE loss: $\rho_{S}$ = 0.64, p = 0.001; weighted CE + Dice loss: $\rho_{S}$ = 0.45, p = 0.09). The combination of the weighted CE and Dice loss components achieved higher SI values and this case appears to be less affected by lesion load, as indicated by the slightly lower correlation value. This correlation between SI values and lesion load reflects a variation in performance with the amount of WMHs that are present and is undesirable for robust performance. However, the correlation coefficients of the cases with the CE loss component and weighted CE loss component were not significantly higher than the combination case (Fisher z-transformation scores, α = 0.45 and α = 0.72 respectively, where α<0.05 indicates that there is a significant difference).

### 3.3 Effect of model dimension on segmentation performance

**Fig. 7:**
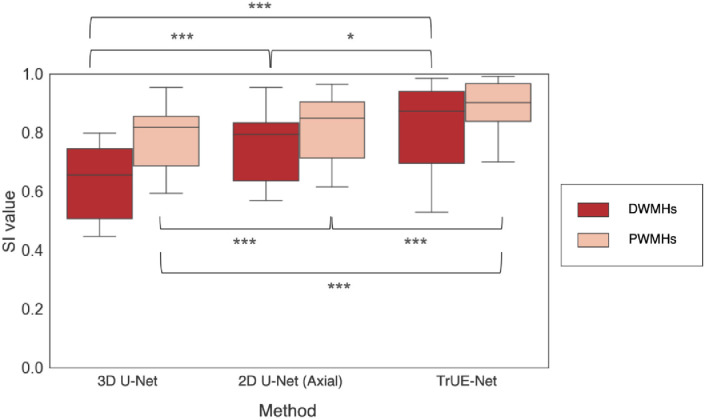
Boxplots of SI values obtained for deep and periventricular regions on the NDGEN dataset for 3D U-Net, 2D U-Net (axial) and TrUE-Net. *** - p < 0.0001, ** - p < 0.001, * - p < 0.01.

The boxplots of the validation SI values for WMH segmentation in deep and periventricular areas for various cases of model dimensions are shown in Fig. 7. On performing non-parametric Friedman test across three different model dimensions (3D U-Net, 2D axial U-Net, TrUE-Net), we found that the SI values significantly increase for DWMHs ($\chi^{2}$ = 29.1, p < 0.0001) and PWMHs ($\chi^{2}$ = 39.9, p < 0.0001) across three different cases from 3D U-Net to TrUE-Net. Both 2D axial U-Net and TrUE-Net give significantly higher SI values than 3D U-Net in the deep and periventricular regions. Also, in both regions, TrUE-Net provided significantly higher SI values compared to 2D axial U-Net, due to the addition of 2D U-Nets in the sagittal and coronal planes in the TrUE-Net architecture.

**Fig. 8:**
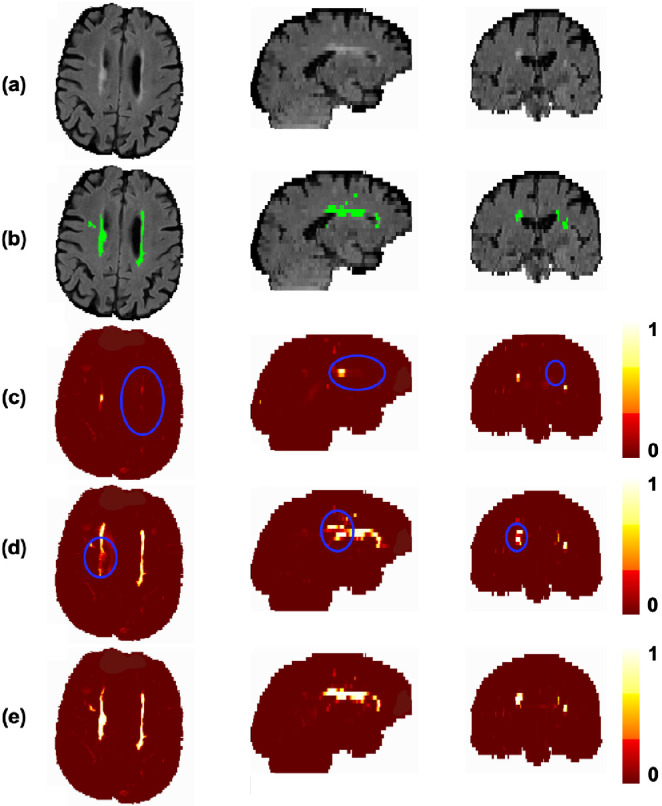
Effect of model dimension on the segmentation in the NDGEN dataset. An example showing (b) manual segmentations against the results of (c) 3D U-Net, (d) 2D axial U-Net and (e) TrUE-Net. Differences in lesion segmentations indicated by blue circles. The colour bar indicates the WMH probability value at each voxel. (For interpretation of the references to colour in this figure legend, the reader is referred to the web version of this article.)

Fig. 8 shows a sample prediction, from all three planes, of our proposed model compared with the 3D U-Net and the 2D axial U-Net. The 3D U-Net detected the most straightforward bright PWMHs, which have high contrast. The model missed most of the DWMHs and provided under-segmentation or incorrect delineation of PWMHs (circled regions in Fig. 8c). We found that the 3D model missed more WMHs as the lesion load decreased, giving a poor segmentation in low lesion load subjects. The 2D axial U-Net gave a better segmentation when compared to the 3D model, but still lacked the contextual information across slices. Hence, it missed parts of WMHs in some of the contiguous slices, leading to discontinuities in WMH detection. An instance of this is shown in Fig. 8d, where the discontinuity is clearly visible in the sagittal and coronal planes. The proposed TrUE-Net model predicted WMHs using contextual information from all three planes and provided a continuous and more accurate segmentation (Fig. 8e and significant improvement in SI values as shown in Fig. 7) using fewer parameters compared to the 3D U-Net.

### 3.4 Evaluation of WMH segmentation performance

#### 3.4.1 Leave-one-out validation

**Fig. 9:**
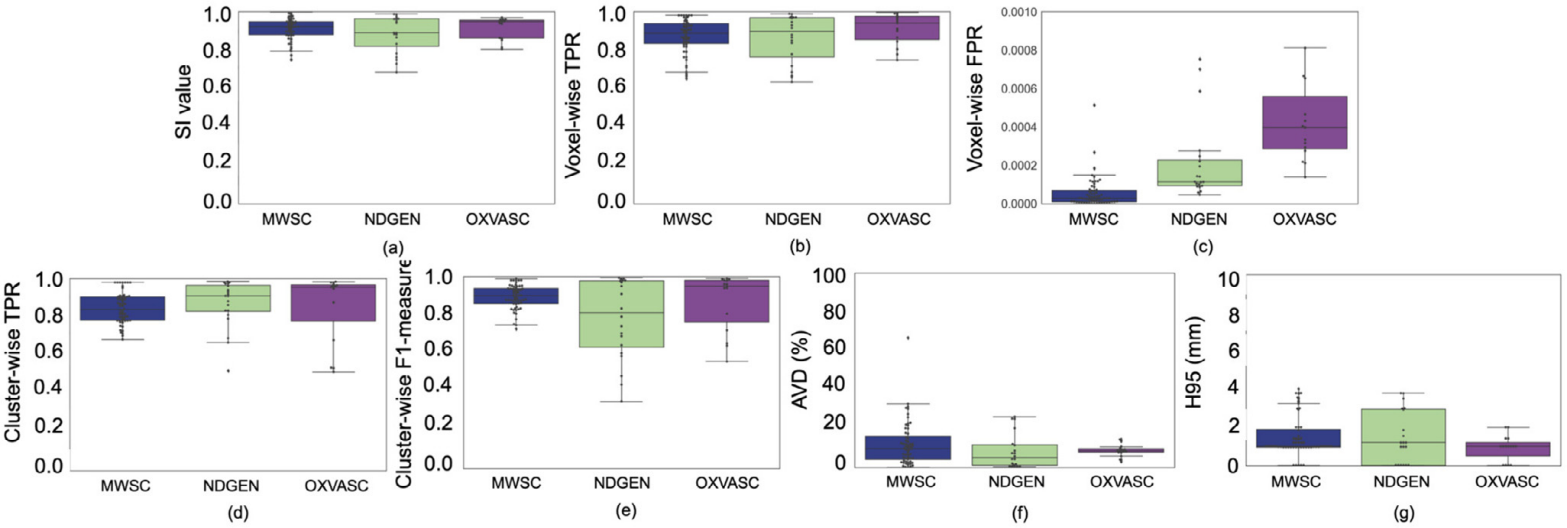
Boxplots of performance metrics obtained from LOO evaluation on the MWSC, NDGEN and OXVASC datasets - (a) SI value, (b) voxel-wise TPR, (c) voxel-wise FPR, (d) cluster-wise TPR, (e) cluster-wise F1-measure, (f) Absolute volume difference (AVD), and (g) 95${}^{\text{th}}$ percentile of Hausdorff distance.

Fig. 9 shows the boxplots of the performance metrics for the leave-one-out (LOO) validation, performed separately, on the MWSC cohorts, NDGEN and OXVASC datasets. Table 1 shows the corresponding values. TrUE-Net achieved its best performance on the MWSC and OXVASC datasets. Within the MWSC cohorts, TrUE-Net achieved the best performance for the Utrecht cohort (SI: 0.92 ± 0.04, voxel-wise TPR: 0.90 ± 0.07, voxel-wise FPR: 0.7 $\times 10^{-4}$, cluster-wise TPR: 0.88 ± 0.08, cluster-wise F1-measure: 0.92 ± 0.07, AVD: 10.95 ± 6.6%, H95: 1.25 ± 0.72 mm). The model achieved the lowest performance on the NDGEN dataset, however with the highest cluster-wise TPR value of 0.87 ± 0.12. This indicates that it detects more true positive lesions when compared to other datasets, while the lesion boundaries were better delineated in other datasets.

**Table 1:** LOO evaluation of TrUE-Net on the MWSC, NDGEN and OXVASC datasets. The median values are provided with the interquartile range (IQR) between 25${}^{\text{th}}$ and 75${}^{\text{th}}$ percentiles reported in parentheses (the best median value for each measure across the datasets is highlighted in bold).

Perf. metrics	MWSC	NDGEN	OXVASC
SI	0.92 (0.88 - 0.95)	0.89 (0.82 - 0.96)	**0.95 (0.86 - 0.96)**
Voxel-wise TPR	0.89 (0.83 - 0.94)	0.89 (0.76 - 0.97)	**0.94 (0.85 - 0.97)**
Voxel-wise FPR	**2.7 (0.9 - 6.8)**$\times 10^{-5}$	1.1 (0.9 - 2.2) $\times 10^{-4}$	3.9 (2.8 - 5.6) $\times 10^{-4}$
Cluster-wise TPR	0.84 (0.78 - 0.90)	0.91 (0.83 - 0.97)	**0.95 (0.78 - 0.97)**
Cluster-wise F1 measure	0.90 (0.86 - 0.94)	0.81 (0.63 - 0.98)	**0.95 (0.76 - 0.98)**
AVD (%)	9.6 (3.9 - 15.9)	**4.9 (0.9 - 11.5)**	8.5 (7.7 - 9.4)
H95 (mm)	**1 (0.96 - 1.89)**	1.2 (0 - 2.94)	**1 (0.5 -1.2)**

**Fig. 10:**
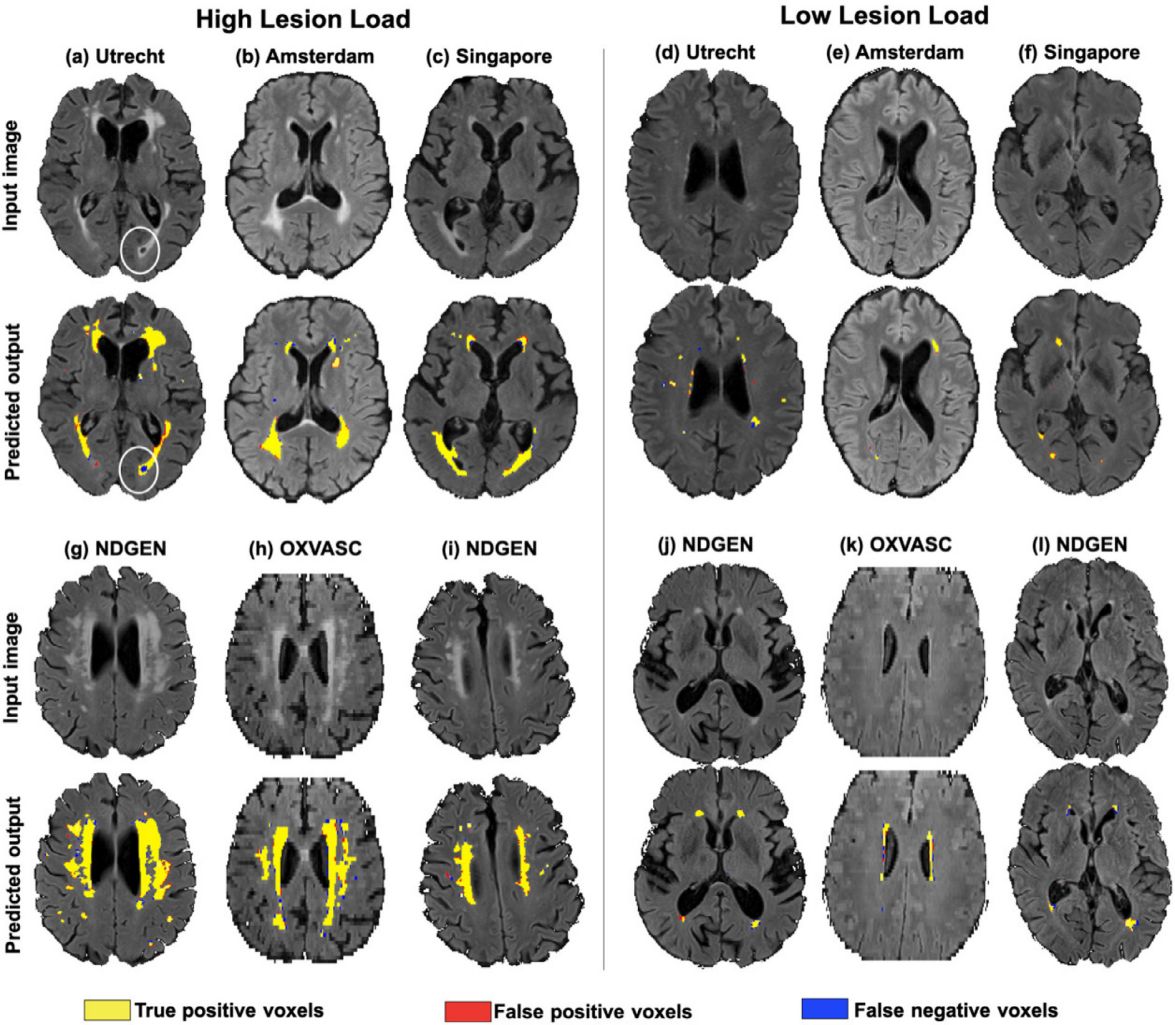
Sample results of TrUE-Net segmentation on the high and low lesion cases - (a, d) Utrecht, (b, e) Amsterdam and (c, f) Singapore cohorts of MWSC dataset, (g, i, j, l) NDGEN and (h, k) OXVASC datasets. True positive, false positive and false negative voxels are indicated in yellow, red and blue respectively. (For interpretation of the references to colour in this figure legend, the reader is referred to the web version of this article.)

The visual results of LOO evaluations on the MWSC cohorts, NDGEN and OXVASC datasets are illustrated by a few examples shown in Fig. 10. In both high and low lesion load cases, TrUE-Net provided an accurate segmentation with respect to manual segmentation without detecting many false positives. In particular, TrUE-Net detected the deep lesions in the low lesion load cases, including subtle ones (Fig. 10f and j). It is also worth noting that, although manual segmentation is used as gold standard for evaluation, there might be inconsistencies and errors that would affect the performance evaluation. For instance, in a high lesion load example from the Utrecht cohort in Fig. 10a, some CSF voxels were included were included in the manual segmentation (indicated by a circle), while TrUE-Net successfully excluded them by providing lower probability values in those regions.

#### 3.4.2 Results on MWSC 2017 unseen test dataset

On evaluating the docker container of our method on the unseen challenge test data, we obtained the following weighted average of performance metrics on the unseen test dataset: SI value - 0.77, H95 - 6.95, AVD - 20.49, cluster-wise TPR - 0.74 and cluster-wise F1 measure - 0.70. For the performance metrics on individual test datasets and the corresponding boxplots, please refer https://wmh.isi.uu.nl/results/fmrib-truenet-2/.

#### 3.4.3 Performance in deep and periventricular regions

**Fig. 11:**
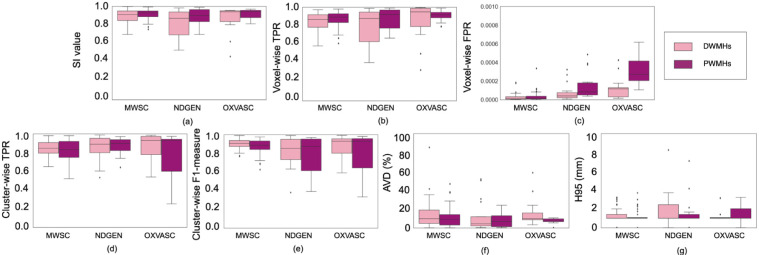
Boxplots of performance metrics obtained in deep and periventricular regions for LOO evaluation on the MWSC, NDGEN and OXVASC datasets - (a) SI value, (b) voxel-wise TPR, (c) voxel-wise FPR, (d) cluster-wise TPR, (e) cluster-wise F1-measure, (f) Absolute volume difference (AVD), and (g) 95${}^{\text{th}}$ percentile of Hausdorff distance.

Fig. 11 shows boxplots of the performance metrics for DWMHs and PWMHs. Table 2 reports the corresponding descriptive statistics, along with the p-values of Wilcoxon signed rank test performed between DWMHs and PWMHs. Most of the performance metrics are not significantly different between PWMHs and DWMHs. Particularly, none of the differences in cluster-wise and voxel-wise TPRs are significant, indicating that TrUE-Net not only successfully detects true lesions in both the periventricular and deep regions, but also segments the lesion boundaries accurately in both regions. The only significant differences can be found in the cluster-wise F1-measure in the MWSC dataset, as well as AVD and voxel-wise FPR values in the MWSC and OXVASC datasets. In the case of the MWSC dataset, the cluster-wise F1-measure in the deep regions were significantly higher than in the periventricular regions. This means that more true DWMHs were detected, with higher cluster-wise precision, compared to PWMHs. In the OXVASC and the MWSC datasets, while DWMHs showed significantly higher AVD percentage values compared to PWMHs, they still correspond to much lower lesion volumes when compared to PWMHs. For instance, AVD value of 25% in the deep region corresponds to a lesion volume of around 600 mm^3^ while the same value corresponds to a much higher value, of around 3800 mm^3^, in the periventricular region.

**Table 2:** Comparison of performance metrics between PWMHs and DWMHs, along with p-values of Wilcoxon signed rank test results on the MWSC, NDGEN and OXVASC datasets (median and IQR values provided; significant p-values highlighted in bold).

		MWSC	NDGEN	OXVASC
SI	DWMHs	0.91 (0.85 - 0.95)	0.87 (0.69 - 0.94)	0.94 (0.84 - 0.95)
	PWMHs	0.93 (0.88 - 0.95)	0.90 (0.84 - 0.97)	0.95 (0.88 - 0.96)
	p-value	0.06	0.05	0.11
Voxel-wise TPR	DWMHs	0.87 (0.79 - 0.92)	0.88 (0.63 - 0.95)	0.95 (0.79 - 0.99)
	PWMHs	0.89 (0.84 - 0.93)	0.92 (0.77 - 0.97)	0.92 (0.89 - 0.95)
	p-value	0.08	0.10	0.45
Voxel-wise FPR	DWMHs	1.3 (0.4 - 3.1) $\times 10^{-5}$	3.8 (2.0 - 7.5) $\times 10^{-5}$	1.1 (0.2 - 1.3) $\times 10^{-4}$
	PWMHs	1.5 (0.6 - 3.7) $\times 10^{-5}$	8.2 (5.0 - 17.9) $\times 10^{-5}$	2.7 (0.2 - 4.7) $\times 10^{-4}$
	p-value	**0.04**	0.06	<**0.001**
Cluster-wise TPR	DWMHs	0.85 (0.79 - 0.91)	0.89 (0.80 - 0.95)	0.93 (0.78 - 0.97)
	PWMHs	0.83 (0.75 - 0.92)	0.90 (0.82 - 0.94)	0.93 (0.60 - 0.94)
	p-value	0.17	0.69	0.25
Cluster-wise F1-measure	DWMHs	0.91 (0.87 - 0.94)	0.85 (0.73 - 0.91)	0.93 (0.80 - 0.95)
	PWMHs	0.89 (0.84 - 0.93)	0.87 (0.61 - 0.95)	0.93 (0.64 - 0.96)
	p-value	**0.02**	0.08	0.17
AVD (%)	DWMHs	9.7 (4.8 - 18.8)	4.1 (1.9 - 11.8)	9.7 (8.6 - 15.7)
	PWMHs	9.0 (2.9 - 14.0)	6.7 (1.2 - 12.9)	7.3 (6.5 - 9.5)
	p-value	**0.002**	0.35	**0.01**
H95 (mm)	DWMHs	1 (1 - 1.41)	1 (1 - 2.45)	1 (1 - 1.7)
	PWMHs	1 (1 - 1.7)	1 (1 - 1.41)	1 (1 - 2.0)
	p-value	0.39	0.19	0.41

Overall, TrUE-Net provided performance metrics for DWMHs on par with PWMHs. This shows that the model provides good delineations of WMHs, with good sensitivity and specificity in both regions.

### 3.5 Comparison with BIANCA

**Fig. 12:**
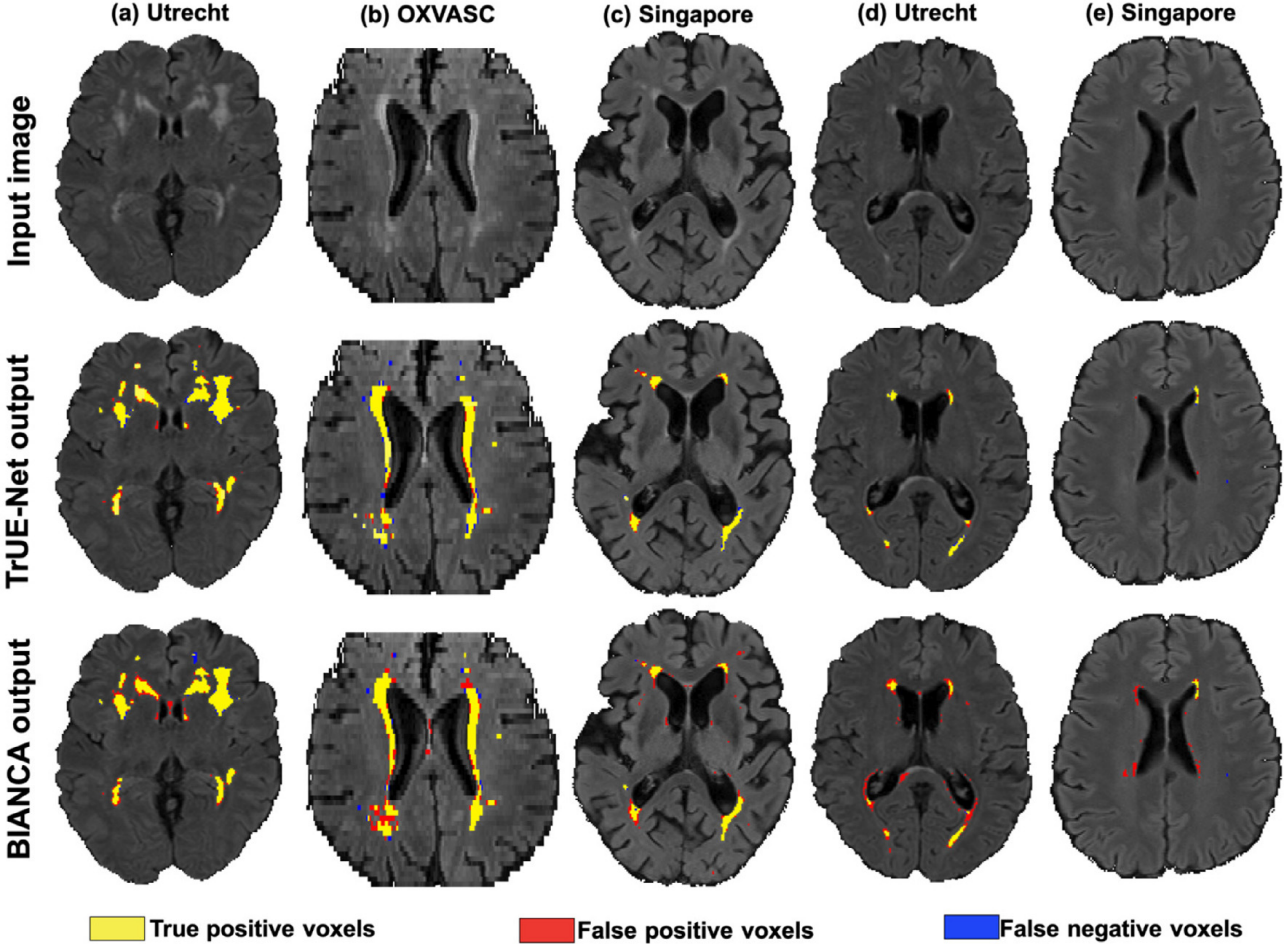
Sample results for comparison of TrUE-Net segmentation with those of BIANCA. Left to right: decreasing order of lesion load from the Utrecht (a,d), Singapore (c, e) and OXVASC (b) datasets. True positive, false positive and false negative voxels are indicated in yellow, red and blue colour respectively. (For interpretation of the references to colour in this figure legend, the reader is referred to the web version of this article.)

Fig. 12 illustrates a few example segmentations obtained with TrUE-Net and BIANCA, with respect to manual segmentation, in the order of decreasing lesion load. TrUE-Net provided more accurate segmentations than BIANCA, especially in the low lesion load subjects. From the figure it can be observed that, as the lesion load decreases, BIANCA detected more false positives, particularly around the ventricles (shown in Fig. 12a, d and e) and oversegmented PWMHs (Fig. 12b and c).

**Fig. 13:**
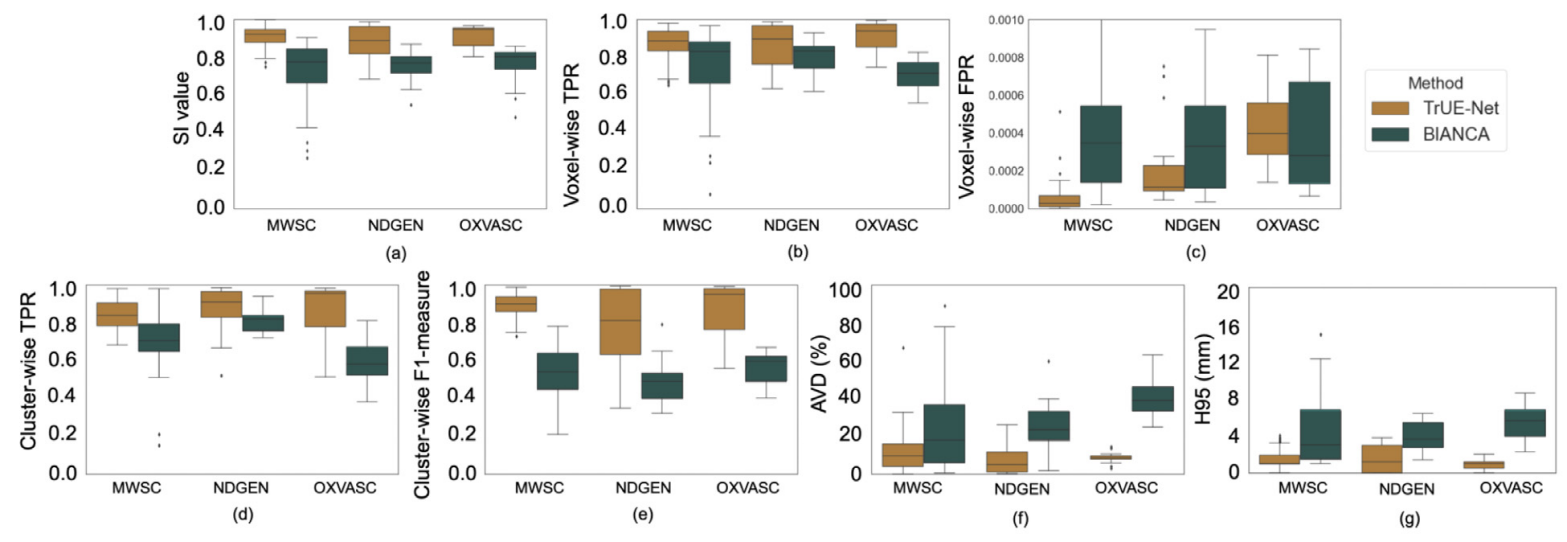
Boxplots of performance metrics obtained for TrUE-Net and BIANCA on the Utrecht, Singapore, Amsterdam, NDGEN and OXVASC datasets - (a) SI value, (b) Absolute volume difference (AVD), (c) voxel-wise TPR, (d) voxel-wise FPR, (e) cluster-wise TPR, (f) cluster-wise F1-measure and (g) 95${}^{\text{th}}$ percentile of Hausdorff distance.(For interpretation of the references to colour in this figure legend, the reader is referred to the web version of this article.)

**Fig. 14:**
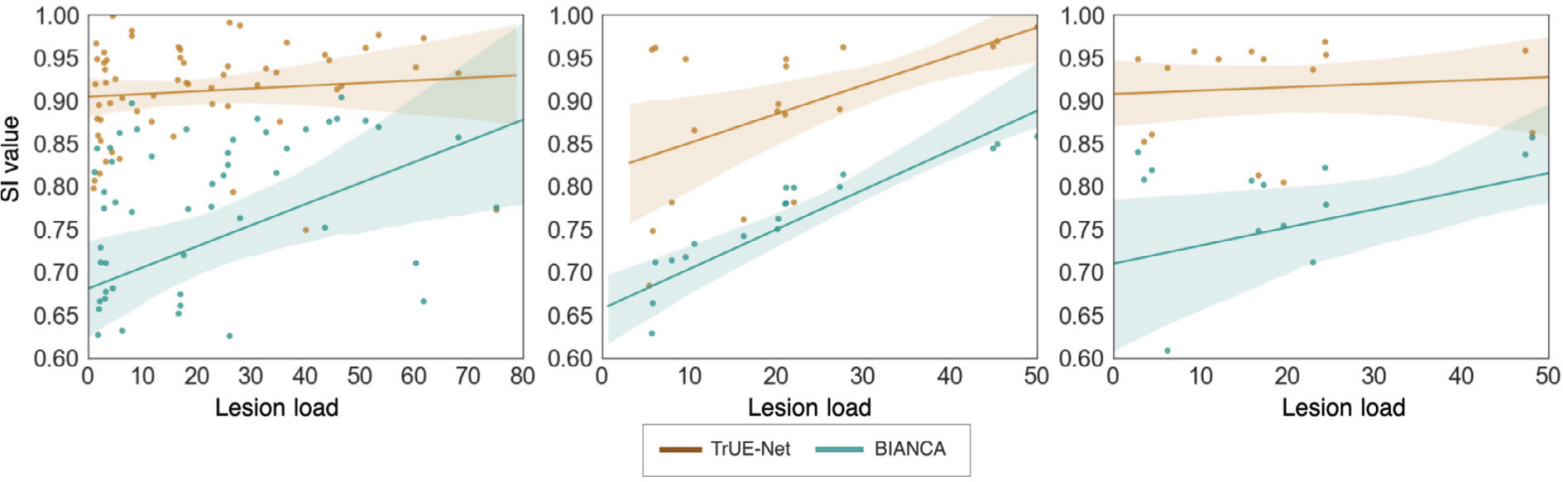
Regression plot of SI values with respect to lesion load for TrUE-Net (brown) and BIANCA (green) on the MWSC, NDGEN and OXVASC datasets. The shaded region represents the 95% confidence interval of the regression estimates. (For interpretation of the references to colour in this figure legend, the reader is referred to the web version of this article.)

Overall, TrUE-Net outperforms BIANCA in both voxel-wise and cluster-wise metrics. Fig. 13 shows the boxplots comparing the performance metrics between TrUE-Net and BIANCA. The corresponding performance values and p-values are reported in Table 3. TrUE-Net provides significantly better results than BIANCA for almost all the performance metrics. BIANCA provided the worst performance on the MWSC dataset, with both more false positives and false negatives than TrUE-Net. In the NDGEN dataset, the voxel-wise TPR values are not significantly different for TrUE-Net and BIANCA, indicating that the two methods performs equally on this dataset. Also voxel-wise FPR were not significantly different between the two methods in both NDGEN and OXVASC datasets. Fig. 14 shows the regression plots of SI values against lesion loads for the MWSC, NDGEN and OXVASC datasets. We determined the Spearman correlation coefficient of the SI values with lesion loads for the methods on the individual datasets and determined their statistical significance. Both TrUE-Net and BIANCA showed positive correlation with lesion loads with significant correlations on the NDGEN dataset (TrUE-Net: $\rho_{S}$ = 0.64, p = 0.002; BIANCA: $\rho_{S}$ = 0.99, p < 0.0001), with significant difference between their correlations (α < 0.0001). In general, the SI values of TrUE-Net showed less correlation with respect to lesion loads compared to those from BIANCA, indicating a consistent performance for all lesion loads.

**Table 3:** Comparison of TrUE-Net performance with BIANCA, along with p-values of Wilcoxon signed rank test results on the MWSC, NDGEN and OXVASC datasets (median and IQR values reported; significant p-values highlighted in bold).

		MWSC	NDGEN	OXVASC
SI	TrUE-Net	0.92 (0.88 - 0.95)	0.89 (0.82 - 0.96)	0.95 (0.86 - 0.96)
	BIANCA	0.77 (0.66 - 0.85)	0.77 (0.72 - 0.80)	0.80 (0.73 - 0.82)
	p-value	≪**0.001**	**0.001**	<**0.001**
Voxel-wise TPR	TrUE-Net	0.89 (0.83 - 0.94)	0.89 (0.76 - 0.97)	0.94 (0.85 - 0.97)
	BIANCA	0.83 (0.66 - 0.88)	0.83 (0.74 - 0.86)	0.74 (0.65 - 0.78)
	p-value	≪**0.001**	0.15	<**0.001**
Voxel-wise FPR	TrUE-Net	2.7 (0.9 - 6.8) $\times 10^{-5}$	1.1 (0.9 - 2.2) $\times 10^{-4}$	3.9 (2.8 - 5.6) $\times 10^{-5}$
	BIANCA	3.4 (1.4 - 5.4) $\times 10^{-4}$	3.2 (1.0 - 5.4) $\times 10^{-4}$	2.6 (1.2 - 6.7) $\times 10^{-4}$
	p-value	≪**0.001**	0.07	0.98
Cluster-wise TPR	TrUE-Net	0.84 (0.78 - 0.90)	0.91 (0.83 - 0.97)	0.96 (0.78 - 0.97)
	BIANCA	0.70 (0.65 - 0.79)	0.82 (0.76 - 0.84)	0.58 (0.55 - 0.68)
	p-value	≪**0.001**	0.09	<**0.001**
Cluster-wise F1-measure	TrUE-Net	0.90 (0.86 - 0.94)	0.81 (0.63 - 0.98)	0.95 (0.76 - 0.98)
	BIANCA	0.54 (0.45 - 0.64)	0.49 (0.40 - 0.53)	0.60 (0.52 - 0.63)
	p-value	≪**0.001**	<**0.001**	<**0.001**
AVD (%)	TrUE-Net	9.6 (3.9 - 15.9)	4.9 (0.9 - 11.5)	8.5 (7.7 - 9.4)
	BIANCA	17.9 (5.9 - 36.7)	23.3 (18.1 - 32.9)	38.5 (33.1 - 48.4)
	p-value	≪**0.001**	<**0.001**	<**0.001**
H95 (mm)	TrUE-Net	1 (0.9 - 1.9)	1.2 (0 - 2.94)	1 (0.5 - 1.2)
	BIANCA	3.0 (1.5 - 6.8)	3.6 (2.7 - 5.4)	5.5 (3.7 - 7.2)
	p-value	≪**0.001**	<**0.001**	<**0.001**

### 3.6 Comparison with the top-ranking method of MWSC 2017

**Fig. 15:**
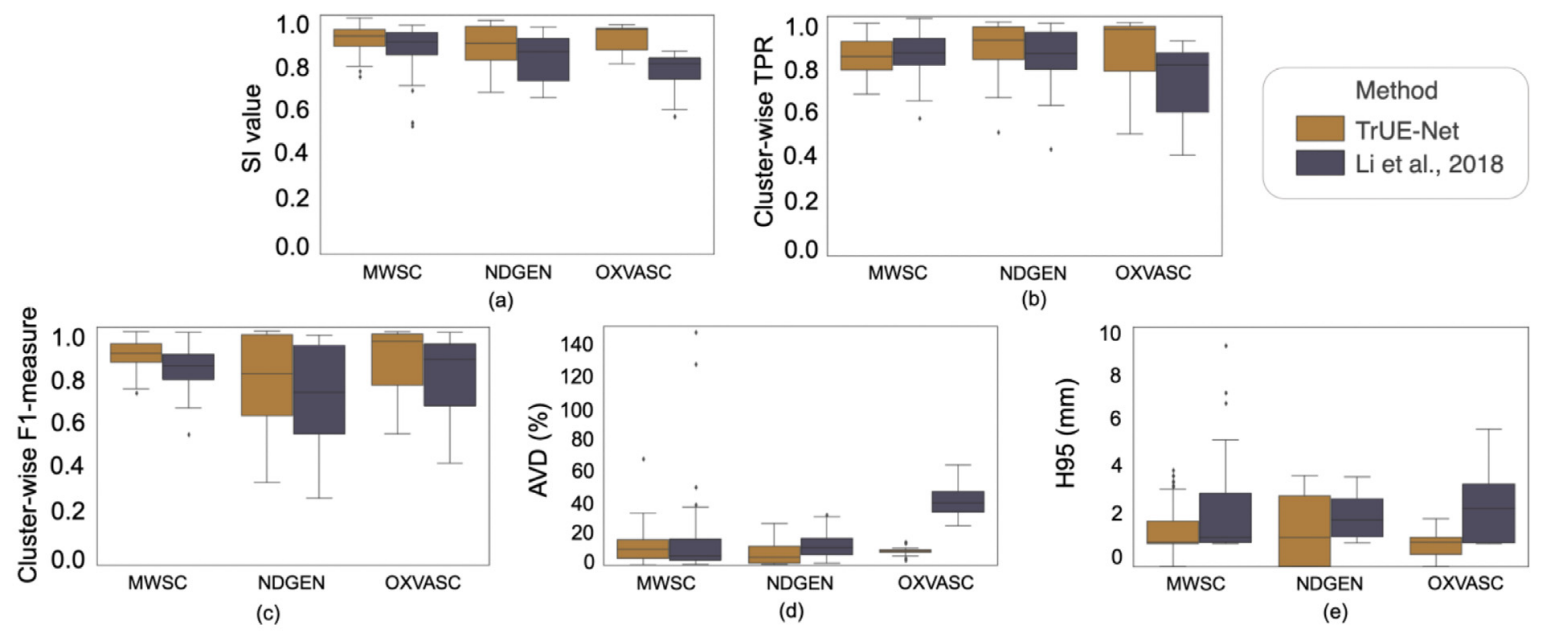
Boxplots of performance metrics obtained for TrUE-Net and Li et al. (2018) on the MWSC dataset - (a) SI value, (b) Cluster-wise TPR, (c) Cluster-wise F1-measure, (d) Absolute volume difference (AVD) and (e) 95${}^{\text{th}}$ percentile of Hausdorff distance. (For interpretation of the references to colour in this figure legend, the reader is referred to the web version of this article.)

Fig. 15 shows the boxplots comparing the LOO performance metrics between TrUE-Net and the method proposed in Li et al. (2018) trained and evaluated separately on the MWSC, NDGEN and OXVASC datasets. The corresponding values and p-values of Wilcoxon signed rank test are reported in Table 4. On the MWSC dataset, TrUE-Net achieves significantly higher SI values and significantly lower H95 values compared to Li et al. (2018). However, Li et al. (2018) achieves better cluster-wise TPR values indicating that the method detects more true-lesions compared to TrUE-Net. On the other hand, the cluster-wise F1-measure value is significantly higher for TrUE-Net, which shows that Li et al. (2018) also detects more false positive clusters, while TrUE-Net provides more cluster-wise precision, resulting in a higher cluster-wise F1-measure value. The performance metrics obtained for Li et al. (2018) on the NDGEN dataset were significantly lower (except H95) compared to those of TrUE-Net. Li et al. (2018) gave the worst performance on the OXVASC dataset, with significantly lower performance metrics when compared to TrUE-Net. This could be due to the fact that the OXVASC dataset consists of lower resolution in the axial plane, which is quite different from the other two datasets. We observed that Li et al. (2018) missed a few small lesions and undersegmented the lesions, missing voxels along the lesion boundaries.

**Table 4:** Comparison of TrUE-Net performance with Li et al. (2018), along with p-values of Wilcoxon signed rank test results on the MWSC, NDGEN and OXVASC datasets (median and IQR values reported; significant p-values highlighted in bold).

		MWSC	NDGEN	OXVASC
SI	TrUE-Net	0.92 (0.88 - 0.95)	0.89 (0.82 - 0.96)	0.95 (0.86 - 0.96)
	Li et al., 2018	0.90 (0.84 - 0.94)	0.85 (0.73 - 0.91)	0.80 (0.73 - 0.82)
	p-value	**0.003**	<**0.001**	<**0.001**
Cluster-wise TPR	TrUE-Net	0.84 (0.78 - 0.90)	0.91 (0.83 - 0.97)	0.96 (0.78 - 0.97)
	Li et al., 2018	0.85 (0.80 - 0.91)	0.85 (0.79 - 0.94)	0.82 (0.68 - 0.85)
	p-value	0.49	**0.003**	<**0.001**
Cluster-wise F1-measure	TrUE-Net	0.90 (0.86 - 0.94)	0.81 (0.63 - 0.98)	0.95 (0.76 - 0.98)
	Li et al., 2018	0.84 (0.79 - 0.89)	0.73 (0.56 - 0.93)	0.89 (0.71 - 0.94)
	p-value	<**0.001**	<**0.001**	**0.003**
AVD (%)	TrUE-Net	9.6 (3.9 - 15.9)	4.9 (0.9 - 11.5)	8.5 (7.7 - 9.4)
	Li et al., 2018	5.3 (2.8 - 16.2)	10.9 (6.5 -16.4)	38.5 (33.1 - 48.4)
	p-value	0.48	<**0.001**	<**0.001**
H95 (mm)	TrUE-Net	1 (0.9 - 1.9)	1.2 (0 - 2.94)	1 (0.5 - 1.2)
	Li et al., 2018	1.2 (1 - 3.0)	1.9 (1.2 - 2.8)	2.4 (1.2 - 3.4)
	p-value	**0.009**	0.25	<**0.001**

### 3.7 Comparison with other existing methods

In order to contextualise the impact of the methods/improvements presented in this work, Table 5 illustrates an indirect comparison with other existing methods in the literature. The SI values obtained with TrUE-Net are higher than those reported for the non-DL methods in the existing literature, including BIANCA. In the case of DL methods, the performance of TrUE-Net is comparable to the top-performing DL methods in the challenge on the unseen test dataset.

**Table 5:** Comparison of existing methods (including BIANCA) with TrUE-Net^a^.

Method	Type^b^	Population - study^c^ (subjects)	Image modalities	SI value	Sens^d^	Spec^e^
Wang et al. (2012)	I,U	Singapore ageing cohort (272)	T1, T2, FLAIR	0.77	0.81	0.97
Gibson et al. (2010)	I,U	WM disease (18)	FLAIR	0.81	-	-
De Boer et al. (2009)	I,S	HC - Rotterdam scan study(6)	T1, PD, FLAIR	0.72	0.79	-
Steenwijk et al. (2013)	I,S	Hypertension (20)	T1, FLAIR	0.84	-	-
Damangir et al. (2012)	IA,S	HC, Alzheimer’s, dementia - DemWest (102)	T1, FLAIR	-	0.90	0.99
Ghafoorian et al. (2016)	IA,S	Small vessel disease dataset (50)	PD, T2, FLAIR	-	0.73	-
Yoo et al. (2014)	I,S	Longitudinal/ dementia - Korean study (32)	FLAIR	0.76	-	-
Jeon et al. (2011)	IA,U	SVD - AMPETIS (45)	PD, T2, FLAIR	0.90	-	-
Yang et al. (2010)	IA,U	Dementia - LEILA (30)	FLAIR	0.81	-	-
Shi et al. (2013)	IAAp,U	Acute infarction (91)	DWI, T1, FLAIR	0.84	0.80	-
Samaille et al. (2012)	IAAp,U	MCI, CADASIL (67)	T1, FLAIR	0.72	-	-
Kruggel et al. (2008)	IAAp	HC, dementia - LEILA (116)		-	0.90	0.91
Khademi et al. (2011)	IAAp,U	Subjects with lesions (24)	FLAIR	0.83	0.82	0.99
Admiraal-Behloul et al. (2005)	IA,U	Vasc. disease - PROSPER (100)	PD, T2, FLAIR	0.75	-	-
Anbeek et al. (2004)	IA,S	Arterial vascular disease (20)	T1, IR, PD, T2, FLAIR	0.80	0.97	0.97
Li et al. (2018)	DL	MWSC TS	T1, FLAIR	0.80	-	-
Andermatt et al. (2016)	DL	MWSC TS	T1, FLAIR	0.78	-	-
Berseth, 2017	DL	MWSC TS	T1, FLAIR	0.77	-	-
Valverde et al. (2017)	DL	MWSC TS	T1, FLAIR	0.77	-	-
Kuijf et al. (2019)	DL	MWSC TS	T1, FLAIR	0.77	-	-
Kuijf et al. (2019)	DL	MWSC TS	T1, FLAIR	0.72	-	-
Xu et al. (2017)	DL	MWSC TS	T1, FLAIR	0.73	-	-
Ghafoorian et al. (2017)	DL	Elder SVD (50)	T1, FLAIR	0.78	-	-
BIANCA (Griffanti et al., 2016)	IAAp,S	NDGEN (21)	T1, FLAIR	0.77	0.80	-
		OXVASC (18)	T1, FLAIR, MD	0.74	0.71	-
		MWSC TrS (60)	T1, FLAIR	0.73	0.74	-
TrUE-Net	DL	MWSC TrS (60)	T1, FLAIR	0.91	0.87	-
		NDGEN (20)	T1, FLAIR	0.88	0.86	-
		OXVASC (18)	T1, FLAIR	0.91	0.91	-
TrUE-Net	DL	MWSC TS	T1, FLAIR	0.77	-	-

a Mean values of evaluation metrics reported for all methods (including TrUE-Net).

b Type: I - intensity, IA - intensity + anatomy, IAAp - intensity + anatomy + appearance, U - unsupervised, S - supervised, DL - deep learning

c Population-study: MWSC TS - MICCAI WMH segmentation Challenge test dataset, MWSC TrS - MICCAI WMH segmentation Challenge training dataset (Kuijf et al., 2019)

d Sensitivity or Voxel-wise TPR,

e Specificity.

## 4 Discussion and conclusions

In this work, we proposed a DL model using an ensemble of U-Nets, named TrUE-Net, for accurate WMH segmentation. First, we investigated the effect of various training hyperparameters on model optimisation. We then studied the effect of various components of the loss function and of the model dimension on the segmentation performance. On data from 5 cohorts, we evaluated the overall segmentation performance as well as the performance in deep and periventricular regions separately. In addition, we directly compared our method with a non-DL method (BIANCA) and a DL method (the top ranking method of MWSC 2017). Finally, we provided an the indirect comparison with various methods proposed in the literature.

When optimising our model, we observed that using a lower batch size resulted in noisy but lower loss values. The lower batch size could be advantageous due to two reasons: lower computation load per batch (hence higher speed) and faster convergence due to the regularisation effect of noisy gradient estimation (Wilson, Martinez, 2003, Keskar, Mudigere, Nocedal, Smelyanskiy, Tang, 2016). Firstly, using smaller batches reduces the gradient estimation time per iteration. However, this would increase the overall number of iterations per epoch, which brings us to the second advantage. Due to the noisy estimate of mean gradient over batches, during subsequent iterations in our experiments we observed that the cost function fluctuates, getting out of some spurious local minima and converging to a better local minima quickly when using smaller batches. Additionally, in our experiments smaller batch sizes avoided over-fitting, as evident from the lower validation loss for a batch size of 8, compared to 16 and 32 (Fig. 3a). Regarding the parameter ϵ, we chose the value 1 $\times 10^{-4}$ as an optimal value for further experiments, since it showed lower loss values. The parameter ϵ is used in the denominator (to avoid divide-by-zero error) in the determination of updates for weights. Having a very low ϵ value results in estimation of larger weight updates, leading to unstable optimisation as shown in the case of 1 $\times 10^{-6}$ (Fig. 3b). In the case of learning rate, choosing a higher value results in earlier convergence with higher loss values. On the other hand, lower values require more epochs to converge due to smaller steps of the updates in weights. Hence, we chose an optimal learning rate schedule between 1 $\times 10^{-3}$ and 1 $\times 10^{-6}$, Fig. 3c, for our further experiments.

When comparing the results using different components of the loss function, we observed that the CE loss component is responsible for identification of the majority of lesions against the background voxels, while the Dice loss component is more sensitive to smaller lesions and precise boundaries. Using the Dice loss component individually provided significantly lower SI values when compared to the case of combined loss function, and provided results comparable to similar methods using Dice loss function for other segmentation applications (Piantadosi et al., 2020). While PWMHs and DWMHs differ in many aspects, like intensity, contrast and texture, these information are learnt by the model while training. Therefore, we used distance values (combination of distances from ventricles and GM) as an additional anatomical prior, derived in a data-driven manner, for weighting the CE loss function to make WMH segmentation better in the deep region. Also, these distance values are independent of lesion load and characteristics and depend only on anatomical structures such as ventricles and GM, which makes the segmentation better irrespective of lesion load. In addition, using the Dice loss component also results in the detection of more subtle deep lesions, giving more accurate segmentation (Fig. 5e) and avoiding over-segmentation in low lesion load subjects. This results in higher SI values in these low lesion load subjects (shown in Fig. 6). Regarding the effect of model dimension, the 3D U-Net model detects most of the PWMHs lesions, since they are larger and have more contextual information, but it failed to detect smaller DWMHs (Figs. 8c and 7). The 2D model is more suitable for those cases, but the 2D model in a single plane alone is not sufficient to capture contextual information across slices and hence often misses lesions in contiguous slices, resulting in discontinuous segmentation (Fig. 8d). Hence, the triplanar ensemble of 2D U-Nets are better than a single plane 2D U-Net since the ensemble model has lower bias towards spurious detections compared to its constituent single-plane models. Moreover, the triplanar model sees data from different planes, thereby avoiding discontinuities in WMHs across consecutive slices and attenuating noise voxels. Hence using the triplanar model leads to both sensitive and precise detection (Figs. 8e and 7), with fewer parameters than the 3D U-Net model.

The TrUE-Net model gave good performance in all 5 datasets. In particular, in the MWSC dataset, TrUE-Net achieved the best cluster-wise F1-measure and the lowest voxel-wise FPR indicating precise and accurate segmentation. The MWSC cohort consists of subject with variations in lesion load and acquisition characteristics. Even though the SI value on the NDGEN dataset is lower than the other two datasets, TrUE-Net detects more small true lesions (best cluster-wise TPR) compared to the other datasets.

When comparing the performance of our model in the deep and the periventricular regions, the trend of performance metrics for DWMHs and PWMHs remained consistent across datasets. In general, we observed that SI values and voxel-wise TPR values were higher for PWMHs when compared to DWMHs. In the deep regions, cluster-wise TPR was higher than that in the periventricular regions. This means that more DWMHs are detected correctly (also evident from higher cluster-wise F1-measure values). However, the lesion boundaries are delineated better in PWMHs when compared to DWMHs, as indicated by slightly higher Hausdorff distance for DWMHs (Fig. 11 and Table 2). At this point, it is worth remembering that manual segmentation is our gold standard but not necessarily the absolute truth. Therefore these errors in the delineation of DWMHs could be due to inconsistencies in manual segmentation (due to low contrast and other confounders) rather than lower model sensitivity. Moreover, the application of the white matter mask in the post-processing step might affect the segmentation of DWMHs near the WM-GM interface. However, when specifically testing this on our data, we observed that differences in the performance metrics near the WM-GM interface with and without applying the WM mask were negligible and not significant (for more details refer to the supplementary material).

On comparing TrUE-Net with BIANCA, we found that TrUE-Net outperforms BIANCA with significant differences in the performance metrics in almost all datasets. Overall, we found that TrUE-Net achieves the highest SI values, cluster-wise F1-measures and the lowest Hausdorff distance values for all datasets, and provides better segmentation in various lesion load cases (Fig. 13). TrUE-Net performs well even in subjects with low lesion load, detecting fewer false positives than BIANCA. Moreover, the SI values achieved by TrUE-Net are higher and less affected by lesion loads when compared to BIANCA, especially on the MWSC and OXVASC datasets as shown in Fig. 14. This shows that, not only with high lesion loads, but also in the cases of small lesion loads where the lesions are quite small and looks like normal ventricle lining (Fig. 12e), TrUE-Net detects the lesions more accurately when compared to BIANCA. This is likely due to the fact that TrUE-Net learns the overall contextual information regarding the lesion distribution. On the other hand, BIANCA uses hand-crafted features (e.g. intensity) that might be affected by contrast/texture variations, thus leading to a noisy segmentation. Among our validation datasets, only the MWSC dataset includes subjects with very low WMH load so further evaluations on bigger samples of low lesion data will be needed to generalise the performance of TrUE-Net. However, this comparison against BIANCA shows promising improvement in the low lesion load range. Also, TrUE-Net provides results comparable with the top ranking method of the MICCAI Challenge (Li et al. (2018)) on all datasets, with significantly higher mean SI values on the MWSC and OXVASC dataset. Particularly, the OXVASC dataset has lower resolution in the axial plane which is quite different from the characteristics of other datasets. TrUE-Net achieved better performance on this dataset due to its triplanar architecture, while Li et al. (2018) provided incorrect segmentation of lesion boundaries, even in PWMHs, due to lower axial resolution. When performing an indirect comparison of TrUE-Net with other existing methods, we observed that only a few methods have been evaluated on a variety of datasets (pathological population and/or healthy subjects). Most of the methods considered for the comparison analysis are tested on datasets from a specific population, and hence might require additional experimentation to validate/improve their generalisability (e.g. fine-tuning of parameters, change of cut-off/threshold values). Some of the multimodal methods in the literature allow the flexibility of choosing different input modalities, while others use fixed sets of modalities. There are both pros and cons associated with using either of the methods. Methods that are flexible allow users to use various available modalities and could handle datasets with missing modalities. On the other hand, the choice of input modalities becomes an additional parameter to tune when applied on an unknown dataset. Also, from our comparison analysis we observed that the use of more modalities might not always lead to better performance. For instance, for the OXVASC dataset, the best performance for BIANCA was achieved with T1 + FLAIR + MD, while TrUE-Net provided better SI values by using only T1 + FLAIR.

The results of evaluation of TrUE-Net on the unseen MWSC test datasets show that TrUE-Net performs well on data from different scanners, and in fact provides consistently high SI values, even for two additional datasets from unseen scanners (VU Amsterdam 3T Philips Ingenuity and VU Amsterdam 1.5T GE Signa HDxt). However, we observed that the cluster-wise performance metrics were lower for TrUE-Net when compared to the top ranking methods (table II in Kuijf et al. (2019)), despite the high SI values (https://wmh.isi.uu.nl/results/fmrib-truenet-2/). This shows that while TrUE-Net provides better segmentation of the detected true WMHs, it still misses small and subtle WMHs, especially in the unseen test datasets. Also, to further test generalisability, we trained TrUE-Net on the NDGEN dataset and tested it on the OXVASC dataset (different population, resolution and axis of acquisition). We observed that the NDGEN-trained model provides a segmentation performance comparable with the LOO evaluation on the OXVASC dataset, with significant increase in only voxel-wise FPR and H95 values (for more details and results, refer to supplementary material). Hence, an interesting future direction of research for this work could be to explore various domain adaptation techniques to improve the detection of WMHs across various unseen datasets, with the use of limited training data.

In conclusion, we proposed a model that provides accurate segmentation, with better performance than BIANCA and on par with the top ranking methods of MWSC 2017. We evaluated it on various datasets with different population and lesions characteristics. Regarding the tool availability, the python implementation of the training and evaluation codes for the TrUE-Net tool is currently available in https://www.git.fmrib.ox.ac.uk/vaanathi/truenet and the docker image of our method containing pretrained model (trained on MWSC) submitted to MWSC is available to download from the docker hub (https://hub.docker.com/repository/docker/wmhchallenge/fmrib-truenet_2). Additionally, TrUE-Net will be integrated as an independent WMH segmentation tool in a future release of FSL, with options to facilitate easier training, testing and fine tuning of models on various datasets in a user-friendly manner.

## CRediT authorship contribution statement

**Vaanathi Sundaresan:** Conceptualization, Methodology, Software, Validation, Formal analysis, Investigation, Writing – original draft. **Giovanna Zamboni:** Supervision, Resources, Writing – review & editing. **Peter M. Rothwell:** Resources, Writing – review & editing. **Mark Jenkinson:** Conceptualization, Supervision, Writing – review & editing, Funding acquisition, Project administration. **Ludovica Griffanti:** Conceptualization, Data curation, Supervision, Writing – review & editing, Project administration.

## Declaration of Competing Interest

The authors declare the following financial interests/personal relationships which may be considered as potential competing interests:

Mark Jenkinson receives royalties from licensing of FSL to non-academic, commercial parties.

## Appendix A Supplementary materials

Supplementary Data S1 Supplementary Raw Research Data. This is open data under the CC BY license http://creativecommons.org/licenses/by/4.0/ Supplementary Data S1

## Footnotes

2 A white matter mask was first obtained from a dilated and inverted cortical CSF tissue segmentation using FSL FAST. Then the WM was subtracted from the brain mask to obtain the GM mask.

3 Training code available at: https://github.com/hongweilibran/wmh_ibbmTum
