# Supplementary material for: Triplanar ensemble U-Net model for white matter hyperintensities segmentation on MR images
Source: Med Image Anal. 2021 Oct;73:102184. doi: 10.1016/j.media.2021.102184 (PMC8505759; doi:10.1016/j.media.2021.102184)
Supplement: Supplementary Data S1 — Supplementary Raw Research Data. This is open data under the CC BY license http://creativecommons.org/licenses/by/4.0/ [file mmc1.pdf]

Supplementary material - Triplanar ensemble U-Net model for white matter hyperintensities segmentation on MR images

Effect of post-processing WM mask on WMH segmentation

Segmentation errors in the WM mask (obtained by dilating cortical CSF) might lead to the exclusion of true lesion voxels in the WM-GM interface. Hence, we performed two additional experiments looking at the effect of the post-processing on the overall segmentation and specifically close to the WM-GM interface. In the first experiment we evaluated the WMH segmentation without applying the WM mask in the post-processing step. Table S1 reports the median and interquartile range (IQR) values (in parentheses) of the difference in the metric between the results with and without WM mask. From the table, it can be observed that the difference in the metrics are quite small and we observed that none of the differences are significant (using Wilcoxon signed rank test).

Table S1: Difference in evaluation metric values between the results with and without WM mask in LOO evaluation of TrUE-Net on the MWSC, NDGEN and OXVASC datasets. The median values are provided with the interquartile range (IQR) between 25th and 75th percentiles reported in parentheses.

**Perf. metrics**	**MWSC**	**NDGEN**	**OXVASC**
SI	2.8 (1.5 - 3.5) $\times 10^{-3}$	0.5 (0.3 - 1.6) $\times 10^{-3}$	2.1 (1.2 - 4.2) $\times 10^{-3}$
Voxel-wise TPR	4.9 (2.7 - 8.4) $\times 10^{-4}$	8.0 (3.0 - 11.2) $\times 10^{-4}$	0.8 (0.5 - 3.8) $\times 10^{-4}$
Voxel-wise FPR	0.5 (0.2 - 0.7) $\times 10^{-4}$	0.6 (0.4 - 0.7) $\times 10^{-4}$	3.9 (2.8 - 5.6) $\times 10^{-4}$
Cluster-wise TPR	0.6 (0.3 - 0.8) $\times 10^{-3}$	1.4 (0.9 - 6.5) $\times 10^{-3}$	0.7 (0.4 - 0.9) $\times 10^{-3}$
Cluster-wise F1 measure	3.0 (1.8 - 4.5) $\times 10^{-3}$	0.9 (0.6 - 2.1) $\times 10^{-3}$	0.7 (0.3 - 1.1) $\times 10^{-3}$
AVD (%)	0.7 (0.32 - 1.0)	1.2 (0 - 2.94)	0.5 (0.3 - 1.5)
H95 (mm)	0.25 (0.17 - 0.37)	0.82 (0.34 - 1.84)	0.6 (0.3 - 1.2)

In the second experiment, we determined the false negative ratio (FNR = number of false negative voxels / number of true lesion voxels) for the cases with and without applying WM mask in the area at the interface between WM and GM. We considered the areas farther than 13 mm from ventricles within the brain (as recommended in [Griffanti et al., 2018, Kim et al., 2008]) as possible WM-GM interface. Within this

area, we determined the performance metrics with and without the application of WM mask used in the post-processing step. For both cases, we achieved the FNR values of 0.11 (0.06 – 0.16), 0.10 (0.03 – 0.24) and 0.05 (0.02 – 0.15) for the MWSC, NDGEN and OXVASC datasets respectively (median and IQR values provided). The differences in the FNR values between the two cases are < 0.001, none of them significant.

3-Fold cross validation on the MICCAI WMH Segmentation Challenge (MWSC 2017) dataset

We performed 3-fold cross validation on the MWSC dataset (consisting of 60 subjects) with 32 training, 8 validation and 20 test subjects for each fold. For training, we used the hyperparameters specified in section 2.1.5 in the paper. Table S2 reports the results of the fold validation on the MWSC dataset.

Table S2: Evaluation metric values for the LOO evaluation and 3-fold cross validation on the MWSC dataset. The median values are provided with the interquartile range (IQR) between 25th and 75th percentiles reported in parentheses. P-values of Wilcoxon signed rank test results between the two cases provided with significant differences highlighted in bold.

**Perf. metrics**	**Leave-one-out (LOO) evaluation**	**3-fold cross validation**	**p-value**
SI	0.92 (0.88 - 0.95)	0.91 (0.86 - 0.95)	0.21
Voxel-wise TPR	0.89 (0.83 - 0.94)	0.88 (0.82 - 0.95)	0.93
Voxel-wise FPR	2.7 (0.9 - 6.8) $\times 10^{-5}$	3.3 (1.0 - 7.8) $\times 10^{-5}$	0.81
Cluster-wise TPR	0.84 (0.78 - 0.90)	0.84 (0.76 - 0.91)	0.60
Cluster-wise F1 measure	0.90 (0.86 - 0.94)	0.89 (0.85 - 0.94)	0.54
AVD (%)	9.6 (3.9 - 15.9)	12.2 (11.0 - 14.2)	0.08
H95 (mm)	1 (0.96 - 1.89)	1.18 (0.8 - 1.6)	0.53

From table R3, there are no significant differences between LOO and 3-fold validations on the MWSC dataset. However, the IQR range is slightly wider in the 3-fold validation indicating high variance in the performance metrics in 3-fold validation.

Results of WMH segmentation on the OXVASC dataset using the model trained on the NDGEN dataset

The generalisability across various datasets is one of the desirable attributes of the model. The results on the MWSC 2017 unseen test dataset provides an indication of model generalisability on MWSC dataset. Additionally, we assessed the performance of the model in the case when the model is trained on the NDGEN dataset and tested on the OXVASC dataset. For training, we used the hyperparameters specified in section 2.1.5 in the paper. Table S3 provides the performance on the OXVASC

dataset for LOO evaluation and for the case where the model is trained on the NDGEN dataset.

Table S3: Evaluation metric values for the LOO evaluation and NDGEN-trained model on the OXVASC dataset. The median values are provided with the interquartile range (IQR) between 25th and 75th percentiles reported in parentheses. P-values of Wilcoxon signed rank test results between the two cases provided with significant differences highlighted in bold.

**Perf. metrics**	**Leave-one-out (LOO) evaluation**	**Model trained on the NDGEN dataset**	**p-value**
SI	0.95 (0.86 - 0.96)	0.93 (0.87 - 0.96)	0.37
Voxel-wise TPR	0.94 (0.85 - 0.97)	0.94 (0.85 - 0.96)	0.92
Voxel-wise FPR	3.9 (2.8 - 5.6) $\times 10^{-4}$	4.5 (0.7 - 6.8) $\times 10^{-4}$	$<$ **0.001**
Cluster-wise TPR	0.95 (0.78 - 0.97)	0.92 (0.74 - 0.96)	0.47
Cluster-wise F1 measure	0.95 (0.76 - 0.98)	0.93 (0.70 - 0.98)	0.95
AVD (%)	8.5 (7.7 - 9.4)	8.8 (4.3 - 15.2)	0.40
H95 (mm)	1 (0.5 - 1.2)	1.14 (0.9 - 1.9)	**0.02**

The results show that the NDGEN-trained model provides a segmentation performance comparable with the LOO evaluation on the OXVASC dataset, with significant increase in only voxel-wise FPR and H95 values.

References

[Griffanti et al., 2018] Griffanti, L., M. Jenkinson, S. Suri, E. Zsoldos, A. Mahmood, N. Filippini, C. E. Sexton, A. Topiwala, C. Allan, M. Kivimäki, et al., 2018, Classification and characterization of periventricular and deep white matter hyperintensities on MRI: a study in older adults: Neuroimage, **170**, 174–181.

[Kim et al., 2008] Kim, K. W., J. R. MacFall, and M. E. Payne, 2008, Classification of white matter lesions on magnetic resonance imaging in elderly persons: Biological psychiatry, **64**, 273–280.
